# Non-Extensive Statistical Analysis of Energetic Particle Flux Enhancements Caused by the Interplanetary Coronal Mass Ejection-Heliospheric Current Sheet Interaction

**DOI:** 10.3390/e21070648

**Published:** 2019-06-30

**Authors:** Evgenios G. Pavlos, Olga E. Malandraki, Olga V. Khabarova, Leonidas P. Karakatsanis, George P. Pavlos, George Livadiotis

**Affiliations:** 1 IAASARS, National Observatory of Athens, 15236 Penteli, Greece; 2 Research Team of Chaos and Complexity, Department of Environmental Engineering, Democritus University of Thrace, 67100 Xanthi, Greece; 3 Heliophysical Laboratory, Pushkov Institute of Terrestrial Magnetism, Ionosphere and Radiowave Propagation RAS (IZMIRAN), Troitsk, Moscow 108840, Russia; 4 Department of Electrical Engineering, Democritus University of Thrace, 67100 Xanthi, Greece; 5 Space Science & Engineering, Southwest Research Institute, San Antonio, TX 78238, USA

**Keywords:** solar wind, energetic particles, magnetic islands, non-extensive statistics, non-Gaussian dynamics, Tsallis q-triplet

## Abstract

In this study we use theoretical concepts and computational-diagnostic tools of Tsallis non-extensive statistical theory (Tsallis q-triplet: $q_{sen},\;q_{rel},\;q_{stat}$), complemented by other known tools of nonlinear dynamics such as Correlation Dimension and surrogate data, Hurst exponent, Flatness coefficient, and p-modeling of multifractality, in order to describe and understand Small-scale Magnetic Islands (SMIs) structures observed in Solar Wind (SW) with a typical size of ~0.01–0.001 AU at 1 AU. Specifically, we analyze ~0.5 MeV energetic ion time-intensity and magnetic field profiles observed by the STEREO A spacecraft during a rare, widely discussed event. Our analysis clearly reveals the non-extensive character of SW space plasmas during the periods of SMIs events, as well as significant physical complex phenomena in accordance with nonlinear dynamics and complexity theory. As our analysis also shows, a non-equilibrium phase transition parallel with self-organization processes, including the reduction of dimensionality and development of long-range correlations in connection with anomalous diffusion and fractional acceleration processes can be observed during SMIs events.

## 1. Introduction

In this work, we focus on the study of the statistical characteristics of Energetic Particle Flux Enhancements (EPFEs) observed in November 2007 by the STEREO A spacecraft, in association with areas filled with Small-scale Magnetic Islands (SMIs). SMIs within the period of interest were previously identified and presented by Khabarova et al. [1]. From the statistical point of view, the occurrence of SMIs may be treated in terms of signatures of intermittent turbulence, which has been done by Adhikari et al. [2]. Such an approach describes general characteristics of the Solar Wind (SW) and energetic particle flux variations associated with SMIs. However, there is another approach to the data analysis that can look deeply into the state of plasma experiencing dynamical changes, in particular, particle energization. Here, we utilize the Tsallis q-entropy theory and non-extensive statistical theory to calculate the Tsallis q-triplet parameters which allows us to obtain significant results on the statistical characteristics of the EPFEs. The physical implications of the q-Tsallis parameters for the particle acceleration mechanism are also discussed.

The SW and space plasma in general are a very interesting medium for the applications of complexity theory and non-extensive statistical physics. In the SW, charged particles, interacting self-consistently with electromagnetic fields, as well as the electromagnetic field itself, constitute a self-consistent nonlinear and non-equilibrium complex system. During the last decades, many scientists have been trying to understand the SW plasma dynamics not only through a simple statistical analysis or case studies, but also employing complexity theory. In particular, Burlaga [3,4,5,6] showed that speed fluctuations possess a multifractal structure in recurrent streams measured between 1 AU and 6 AU. Large scale magnetic field fluctuations observed in the outer heliosphere (at ~25 AU) were found to have the multifractal nature too [3]. Small-scale velocity fluctuations were detected near ~8.5 AU, showing a multifractal structure [4]. These findings suggest that SW turbulence comprises a mixture of sheets and space discontinuities of various scales. Variations of different SW characteristics were further studied by many scientists within a concept of intermittent turbulence in the SW plasma [7,8,9,10,11]. Theoretical models aimed at the explanation of the self-similar, multiscale (multifractal) and intermittent Magnetohydrodynamics (MHD) turbulent character of the SW system were also developed in many works [12,13,14,15,16,17,18,19]. The concept of self-organization and low-dimensional chaotic processes occurring in the SW and space plasmas was also advocated in a series of novel studies by Burlaga [3,4,5,6,20,21], Pavlos et al. [22,23,24,25,26,27,28,29], Karakatsanis et al. [30], Burlaga and Forman [31], Burlaga et al. [32], Macek [16,17], Yang [33], Alberti [34].

Tsallis non-extensive statistical theory and Tsallis extension of the Boltzmann-Gibbs (BG) entropy principle to the q-entropy principle [35,36] reveal a strong character of universality concerning non-equilibrium dynamics [25,26,27,28,29,30]. Tsallis q-entropy principle can explain the emergence of a series of new and significant physical characteristics in spatial distributed systems, as happens in the SW system including magnetic-electric field and particles distributed in the physical space. According to Zelenyi and Milovanov [37], such characteristics are as follows: Non-Gaussian statistics and anomalous diffusion processes, strange and fractional dynamics, multifractal, percolating and intermittent turbulence structures, multiscale and long spatio-temporal correlations, fractional acceleration and Non-Equilibrium (quasi)Stationary States (NESS) or non-equilibrium self-organization process and non-equilibrium phase transition and topological phase transition processes. In these terms, the results presented in this work, clearly reveal strong self-organization and the development of macroscopic ordering of energetic ion intensities related to the strengthening of non-extensivity and multifractality during the evolution of the event.

Energetic particles in the heliosphere and magnetosphere pose significant radiation hazards for astronauts in current and future space missions in the solar system (e.g., to Mars) and for communication satellites (e.g., [38,39,40,41]). Studies of their origin and properties are very important for a timely protection of onboard equipment and human resources. Recently, a new paradigm has emerged in which local magnetic structures, namely small-scale magnetic islands (SMIs) in the SW play a significant role in particle acceleration. SMIs are called so in contrast to large-scale magnetic bubbles/clouds within Interplanetary Corona Mass Ejections (ICMEs). Khabarova et al. [1,42,43,44] found that puzzling or atypical energetic particle flux enhancements of a doubtful origin, Atypical Energetic Particle Enhancements (AEPEs), occur in magnetically confined regions that contain SMIs with a typical width of ~0.01 AU or less. Either the Heliospheric Current Sheet (HCS) or Strong Current Sheets (SCSs) of various origins that have equally strong background magnetic fields provide the magnetic confinement of SMIs that experience dynamical merging or contraction. Zank et al. [45,46,47], le Roux et al. [48,49,50,51] introduced theoretically a new mechanism that can energize particles up to several MeV/nuc locally in the SW. If energetic particles are pre-accelerated to keV energies via classical mechanisms, they may be additionally accelerated up to several MeV inside magnetically confined cavities of various origins. Khabarova et al. [1] showed that this may explain puzzling AEPEs occurring far beyond interplanetary shocks, within ICMEs, before approaching Corotating Interaction Regions (CIRs) as well as between CIRs in space. 

In this work, we use diagnostic tools and computational methods based on nonlinear dynamics and complexity theory as well non-extensive statistics developed by Tsallis [36], in order to obtain deeper physical understanding of the SMIs structures developed in the SW plasma in the interplanetary space. In Section 2 and Appendix A, we present a description of theoretical concepts as the basic theoretical framework of data analysis and physical interpretation. In Section 3, we present the methodology of data analysis. In Section 4, we describe the data of experimental Time Series (TMS). In Section 5, we present the data analysis and results. In Section 6, we summarize the physically significant points of data analysis and results. Finally, in Section 7 we discuss theoretically our results and indicate future research as the natural extension of this study.

## 2. Theoretical Framework

Here we present in brief useful theoretical concepts concerning the non-equilibrium: SW plasma dynamics, plasma thermodynamics, and statistical theory helpful for our SW data analysis and interpretation. The development of SW SMIs and multifractal SW structures that can be observed in situ by spacecraft instrumentation constitute an interesting and significant manifestation of non-equilibrium SW plasma self-organization and topological phase transition process. From the physical point of view, the development of SMIs and SW multifractal structures correspond to the development of intermittent turbulence state that includes multi-scale interaction of fields and particles and simultaneous development of numerous nonlinear plasma instabilities [37]. The development of SMIs and SW intermittent turbulence multifractal plasma structures is a self-consistent electrodynamical, thermodynamical, and non-equilibrium statistical-kinetic plasma phenomenon. That is a complex nonlinear plasma phenomenon described by nonlinear dynamics (strange-fractional nonlinear dynamics) [37,52,53,54,55], Tsallis non-extensive statistical theory [36], as well as by fractal topology and strange kinetics percolation theory [37]. The SMIs and SW fractal-multifractal intermittent turbulence structures are NESS caused by plasma nonlinear and non-equilibrium self-organization process, where different plasma instabilities constitute a universal collective mode [37]. From thermodynamical point of view, the plasma NESS correspond to non-equilibrium local maximization of plasma entropy and local minimization of free energy. Also, according to Tsallis non-extensive statistical theory the non-equilibrium entropy function depends upon the real parameter (*q*) which for q≠1 describes the statistical development of long-range correlations underlying the plasma self-organization process [32]. The development of plasma long-range correlations underlies the plasma NESS corresponding to SMIs and SW multifractal–intermittent turbulence plasma structures, which are explained by the Tsallis q-entropy function maximization [56]. Moreover, at NESS Tsallis q-entropy maximization can explain the anomalous diffusion-anomalous random walk (Levy flights) character of magnetic field-energetic particle dynamics [57]. The Tsallis q-triplet parameters can describe basic non-equilibrium and non-extensive plasma processes during SMIs events. 

The main theoretical concept of this study is the physical connection of intermittent SW plasma turbulence during SMIs events and particle acceleration process through fractional-strange dynamics and non-extensive statistical mechanisms [36,37,52,54]. According to this concept the spatial magnetic field intermittent turbulence and the spatial distribution of magnetic field singularities are related with the local sources of hypothesized induced electric field and the local acceleration of particles. In this way, the spatial distribution of magnetic field singularities is mirrored at the spatial fractal-multifractal topology of the magnetic field, which eventually is mirrored at the hypothesized induced electric field and the energetic particle intensities and their spatial distribution. Moreover, the spatial multifractal distribution of magnetic field and energetic particles is mirrored at the magnetic field-energetic particle time series obtained in situ by spacecraft. These time series include information about the spatiotemporal profile of magnetic field and energetic particle fractal-multifractal spatial distributions. Analytical description of non-equilibrium and non-extensive SW dynamics, thermodynamics, statistical mechanics is presented at the Appendix A, where we introduce the reader of this study in the complexity theory. In the Appendix A novel concepts and processes are included such as: Nonlinear and fractional dynamics, Tsallis non-extensive statistical theory, q-entropy principle, self-organization, topological phase transition, intermittent turbulence, non-Gaussian statistics, fractal-multifractal (multiscale) structures, strange dynamics and fractional acceleration, long-range spatiotemporal correlations, anomalous diffusion-random walk, Levy distribution and Levy flights, q-extension of Central Limit Theorem (q-CLT), Tsallis q-triplet.

## 3. Methodology of Data Analysis 

In order to analyze the energetic particle fluxes and their enhancements (EPFEs), as well as the magnetic field intensity obtained by STEREO A spacecraft during SW SMIs event, identified and studied by Khabarova et al. [1] and Adhikari [2], we use the following diagnostic tools:

- Flatness coefficient (F)
- Tsallis q-triplet $\left(q_{sen},\;q_{rel},\;q_{stat}\right)$
- Correlation Dimension (D)
- Hurst exponent (H)

The physical meaning of these parameters is described at the Appendix A. In the following, we present shortly the above parameters and the corresponding methodology of our data analysis.

### 3.1. Flatness Coefficient F

The intermittent nature of the SW plasma dynamics can be investigated through the Probability Density Functions (PDF) of a set of two-point differenced TMS of an original TMS $\delta B_{\tau }\left(t\right)=B\left(t+\tau \right)-B\left(t\right)$, which can be any physical quantity. The coefficient F corresponding to the Flatness values of the two-point difference for the observed TMS is defined as

(1) $$F=\frac{\langle \delta B_{\tau }{\left(t\right)}^{4}\rangle }{{\langle \delta B_{\tau }{\left(t\right)}^{2}\rangle }^{2}},$$

The coefficient *F* for a Gaussian process is equal to 3. Deviation from Gaussian distributions may imply intermittency, as the parameter τ characterizes also the spatial size of the plasma eddies co-moving in the bulk flow, which contribute to the energy cascade process. According to general theory of turbulence, intermittency appears in the heavy tails of the distribution functions as the dynamics in the vortex is non-random, but deterministic. The Flatness coefficient reveals the non-Gaussian character of the statistics but it cannot give more information for the higher than 4 moments of the distribution. In contrast to the Flatness coefficient, the Tsallis q-statistics and the structure function scaling exponents spectrum include much stronger information about the non-Gaussian character of the turbulent state than the Flatness coefficient. That is the application of Tsallis theory can generate the non-Gaussian distribution itself. Moreover, Tsallis theory can be used for the quantitative prediction of the multifractal character of the turbulent state making efficient the comparison of theoretical predictions and experimental estimations.

### 3.2. Tsallis q-Triplet

Tsallis q-triplet $\left(q_{sen},\;q_{rel},\;q_{stat}\right)$ can be estimated as follows:

(a) Index $q_{sen}$

The index $q_{sen}$ correspond to the entropy production of the system and is related to the singularity spectrum, according to the relation:

(2) $$q_{sen}=1-\frac{\alpha_{\max}\;\alpha_{\min}}{\alpha_{\max}-\alpha_{\min}},$$

where $\alpha_{\min}$ and $\alpha_{\max}$ corresponds to zeros of the singularity spectrum f(α) which can be estimated by the experimental TMS [32]. For this reason, we estimate the scaling exponent function $\tau \left(\bar{q}\right)$ by using the relation:

(3) $$\Gamma \left(\bar{q},\;\Delta t\right)=\sum_{}P_{i}{\left(\Delta t\right)}^{\bar{q}}\approx {\left(\Delta t\right)}^{\tau (\bar{q})},$$

where $\Gamma \left(\bar{q},\Delta t\right)$ is the *q*-th order partition function of the experimental TMS $z\left(t_{i}\right)$ and $P_{i}\left(\Delta t\right)$ is the probability coarse-grained weight for time segments $\Lambda_{i}$ of time size Δt of the experimental TMS $z\left(t_{i}\right)$ [58,59]. The experimental estimation of the scaling exponent is used to estimate the generalized dimension spectrum $D_{\bar{q}}$ through the relation:

(4) $$\tau \left(\bar{q}\right)=\left(\bar{q}-1\right)\;D_{\bar{q}},$$

By using $D_{\bar{q}}$ spectrum we estimate the singularity spectrum f(α) using the Legendre transformation:

(5) $$f\left(\alpha \right)=\bar{q}\alpha -\left(\bar{q}-1\right)\;D_{\bar{q}},$$

where $\alpha =\frac{d\tau \left(\bar{q}\right)}{d\bar{q}}$. We must note here, that the Tsallis q-entropy number is a special number corresponding to the extremization of Tsallis entropy of the system, while the $\bar{q}$ describe the range of real values of generalized dimension spectrum $D_{\bar{q}}$.

The degree of multifractality (or width) is given by:

(6) $$\Delta \alpha =\alpha_{\max}-\alpha_{\min},$$

and the degree of asymmetry *A* can be estimated by the relation:

(7) $$A=\frac{\alpha_{0}-\alpha_{\min}}{\alpha_{\max}-\alpha_{0}},$$

In particular, $\alpha_{0}$ corresponds to the largest fractal dimension, which in this case is f(α)=1. It is important to note here that the singularity exponents α of the singularity spectrum f(α) corresponds to the Holder exponent and reveal the intensity of the topological singularity of the phase space as well as how irregular are the physical magnitudes defined in the phase space of the system. The value $\alpha_{0}$, separates the values of α in two distinct intervals, $\alpha <\alpha_{0}$ and $\alpha >\alpha_{0}$ with different physical meaning. In particular, the left part of singularity spectrum f(α) is related with values α lower than the value $\alpha_{0}$, and correspond to the low dimensional regions of the phase space, which is described by the right part of $D_{\bar{q}}$ spectrum. Similarly, the right part of the singularity spectrum f(α) is related with values α higher than $\alpha_{0}$ and correspond to the high dimensional regions of the phase space, which is described by the left part of the curve $D_{\bar{q}}$ of the generalized dimension spectrum. When A>1, then the singular exponents $\alpha <\alpha_{0}$ are more dominant (left skewness). In this case, the low dimensional regions are also dominant over the high dimensional regions. Low dimensional regions are related with small fluctuations in the physical measurements and a strong fractal character as the singular exponents are lower and the discontinuities are stronger too. The opposite scenario occurs, when A<1 (right skewness). This implies the dominance of high dimensional regions of phase space. The values of singular exponents $\alpha >\alpha_{0}$, are more dominant and the evolution of the physical magnitudes is smoother. The case in which A=1, implies the equivalence of low and high dimensional regions in the phase space.

According to these characteristics of f(α) and $D_{\bar{q}}$ spectra, the high dimensional regions of phase space includes smoother fractal topology than the low dimensional regions, where the fractal character is stronger. As we show in the following, low dimensional regions of phase space cause strong fractional acceleration and anomalous diffusion processes of the SW energetic particles system.

(b) p-modeling of multifractality

According to the p-model, the development of intermittent-multifractal turbulent plasma states can be obtained by a typical multiplicative process formally equivalent to a “two-scale Cantor set”, where the probability of visiting and segment of size $l_{1}$ is *p* and for the remaining segment of size $l_{2}$ is 1−p. The generalized dimension spectrum $D_{\bar{q}}$ corresponding to the p-model multiplicative process, for $l_{1}=l_{2}$, is given by the relation

(8) $$D_{\bar{q}}=\log_{2}{\left[p^{\bar{q}}+{\left(1-p\right)}^{\bar{q}}\right]}^{1/\left(1-\bar{q}\right)},$$

where *p* is the p-model parameter. In the case of space filing homogeneous turbulence (Kolmogorov’s K41 theory), the parameter *p* is equal to 1/2. In this case, $dD_{\bar{q}}/d\bar{q}$=0 and $D_{\bar{q}}=c\;\left(D_{\bar{q}}=\pm 1\right)$ [58,60].

(c) Index $q_{stat}$

The Tsallis $q_{stat}$ corresponds to the optimization (maximization) of Tsallis q-entropy. The values of $q_{stat}$ are derived from the observed optimizing Probability Distribution Functions (PDF) according to the Tsallis q-Gaussian distribution:

(9) $$PDF\;\left[\Delta z\right]\equiv A_{q}\;\left[1+\left(q-1\right)\;\beta_{q}\;{\left(\Delta z\right)}^{2}\right]{\;}^{\frac{1}{1-q}},$$

where the coefficients $A_{q}$, $\beta_{q}$ denote the normalization constants and $q\equiv q_{stat}$ is the entropic or non-extensivity factor $\left(q_{stat}<3\right)$ related to the size of the distribution tail. Our statistical analysis is based on the algorithm described by Ferri et al. [59]. We construct the PDF[Δz] which is associated with the first difference $\Delta z=z_{n+1}-z_{n}$ of the experimental TMS, while the Δz range is subdivided into little “cells” (data binning process) of width $\delta_{z}$, centered at $Z_{i}$ so that one can assess the frequency of Δz-values that fall within each cell/bin. The selection of cell size $\delta_{z}$ is a crucial step for the algorithmic process and its equivalent to solving the binning problem: a proper initialization of the cells/bins can speed up the statistical analysis of the data set and lead to convergence to the exact solution. The resultant histogram is being properly normalized and the estimated q-value corresponds to the best linear fitting to the graph $\ln_{q}p\left(z_{i}\right)vsz_{i}^{2}$, where $\ln_{q}p\left(z_{i}\right)$ is the so-called q-logarithm: $\ln_{q}x=\left(x^{1-q}-1\right)/\left(1-q\right)$. Our algorithm estimates correlation coefficient (CC) for each $\delta_{q}=0.01$ step and the best linear fit is considered to be the one that maximizes CC. The obtained $q_{stat}$, corresponding to the best linear fit is then used to compute the equation:

(10) $$G_{q}(\beta ,z)=\frac{\sqrt{\beta }}{C_{q}}e_{q}{}^{-\beta_{z^{2}}},$$

where:

(11) $$C_{q}=\sqrt{\pi }\cdot \Gamma \left(\frac{3-q}{2(q-1)}\right)/\sqrt{q-1}\cdot \Gamma \;\left(\frac{1}{q-1}\right),\;1<q<3,$$

for different β-values. We select the β-value that minimizes the quantity $\underset{i}{\sum }{\left[G_{qstat}\left(\beta z_{i}\right)-p\left(z_{i}\right)\right]}^{2}$. Equation (10) describes to the optimum q-exponential probability distribution function, related with the maximization of Tsallis q-entropy function [32].

(d) Index $q_{rel}$

The $q_{rel}$ index is given by:

(12) $$q_{rel}=\left(s-1\right)/s,$$

where *S* is the slope of the relaxation profile included in the $\ln_{q}C\left(\tau \right)vs\tau$ plotting of the autocorrelation function C(τ) of the experimental signal. Instead of the autocorrelation function C(τ) we can also use the mutual information I(τ) given in Fraser and Swinney [61] by the relation:

(13) $$\begin{array}{l} I(\tau )=-\sum_{x(i)}P\left(x(i)\right)\;\log_{2}P\left(x(i)\right)-\sum_{x(i-\tau )}P\left(x(i-\tau )\right)\;\log_{2}P\left(x(i-\tau )\right)+\\ +\sum_{x(i)}\sum_{x(i-\tau )}P\left(x(i),x(i-\tau )\right)\;\log_{2}P\left(x(i),x(i-\tau )\right) \end{array}$$

The parameter $q_{rel}$ is related with the relaxation time $\tau_{rel}$ according to the q-exponential function $\Omega \left(t\right)≅e_{q_{rel}}^{-t/\tau_{rel}}$ [32]. The relaxation time depends upon the active degrees of freedom of the system. As the dimensionality of the system decreases, the relaxation process becomes slower and slower because the available degrees of freedom for the energy to disperse are fewer. For strong non-extensivity character of the statistics the $q_{rel}$ increases as the system displays long-range correlations and self-organization causing reduction of active degrees of freedom.

### 3.3. Correlation Dimension D and Surrogates Data

In the reconstructed state space, we estimate the correlation integral C(r,m) as function of radius *r* and embedding dimension *m* with parameter *W* of Theiler according to the relation:

(14) $$C(r,m)=\frac{2}{\left(N-W)(N-W-1\right)}\sum_{i=1}^{N}\sum_{j=i+W+1}^{N}\Theta \left(r-\left\|x(i)-x(j)\right\|\right),$$

When the dynamics is nonlinear then it is possible that low-dimensional attracting sets (strange attractors) exist in the state space. In this case the correlation integral reveals power law scaling profile: $C\left(r\right)\sim r^{D}$, where *D* is the mean fractal dimension of the strange attractor. According to Takens [62] the reconstructed m-dimensional state space is an efficient embedding for m≥2D+1. Efficient embedding means topological equivalence between the real and the reconstructed state space [63]. According to these theoretical concepts when there exist a low-dimensional chaotic (strange) attractor of the dynamics, the fractal dimension *D* which corresponds to the saturation value of the slopes $d_{m}$ of the correlation integrals, with the embedding dimension increasing according to the relation: $D=\underset{m\to \infty }{\lim}d_{m}$. The values $d_{m}$ are the scaling exponents of the correlation integral C(r,m) for low values of r (r→0), according to the relation:

(15) $$C(r,m)\sim \;r^{d_{m}},\;\;\;d_{m}=\lim_{r\to 0}\frac{d\left(\ln C(r,m)\right)}{d\left(\ln(r)\right)}\;,$$

According to Theiler [64] the method of surrogate data is used to distinguish between linearity and nonlinearity as well as between chaoticity and pure stochasticity, since a linear stochastic signal can mimic a nonlinear chaotic process after a static nonlinear distortion. Surrogate data are constructed according to Schreiber and Schmitz [65] to mimic the original data, regarding their autocorrelation and amplitude distribution. In particular, the procedure starts with a white noise signal, in which the Fourier amplitudes are replaced by the corresponding amplitudes of the original data. In the second step, the rank order of the derived stochastic signal is used to reorder the original TMS. By doing this, the amplitude distribution is preserved, but the matching of the two power spectra achieved at the first step is altered. The two steps are subsequently repeated several times until the change in the matching of the power spectra is sufficiently small. Surrogate data thus provide the most general type of nonlinear stochastic signals, produced by a nonlinear distortion of a white noise signal that can approach the geometrical or dynamical characteristics of the original data [64]. They can be used for the rejection of every null hypothesis that identifies the observed low dimensional chaotic profile as a purely non-chaotic stochastic linear process. For an extensive description of the nonlinear analysis algorithm see [66,67]. In order to distinguish a nonlinear deterministic process from a linear stochastic one, we use as discriminating statistic a quantity *Q* derived from a method sensitive to nonlinearity, as the Correlation Dimension. The discriminating statistic *Q* is then calculated for the original and the surrogate data and the null hypothesis is verified or rejected according to the value of “sigma” *S*:

(16) $$S=\frac{\mu_{obs}-\mu_{sur}}{\sigma_{sur}},$$

where $\mu_{sur}$ and $\sigma_{sur}$ is the mean and standard deviation of *Q* on the surrogate data and *obs* is the mean of *Q* on the original data. For a single TMS, $\mu_{obs}$ is the single *Q* value [64]. When *S* takes values higher than 2–3 then the probability that the observed TMS does not belong to the same family with its surrogate data is higher than 0.95–0.99, respectively.

### 3.4. Hurst Exponent H

The Hurst exponent related to the fractal dimension (*F_D_*). For self-similar processes, the local properties are reflected in the global ones, resulting in the relationship:

(17) $$F_{D}=m+1-H,$$

between fractal dimension, *F_D_*, and Hurst coefficient, *H*, for a self-similar surface in *m*-dimensional space [68].

More generally, the Hurst exponent is related with: the anomalous diffusion-random walk process, the connectivity index θ of the fractional topology of the phase space of the system dynamics and the fractional derivatives of temporal and spatial changes. For more details, see Appendix A.1.2.

According to Equation (17), small values of Hurst exponent shows a higher fractal dimension. Oppositely, large Hurst exponent shows small fractional dimension. The values of the Hurst exponent range between 0 and 1. A value of 0.5 indicates a true random process (a Brownian TMS). A Hurst exponent value 1/2<H≤1 indicates “persistent behavior” (super-diffusive). Here an increase (decrease) is probably followed by an increase (decrease). A Hurst exponent value 0≤H≤1/2 indicates “anti-persistent behavior” (sub-diffusive). Here an increase (decrease) is probably followed by a decrease (increase). For the estimation of the Hurst exponent in this study we use the Rescaled Range Analysis (R/S) [69].

The Hurst exponent *H*, is defined in terms of the asymptotic behavior of the rescaled range (R/S) as a function of the time span of a TMS as follows:

(18) $$E{\left(R/S\right)}_{n}=\frac{R(n)}{S(n)}=cn^{H},\;\;\;n\to \infty ,$$

where E(R/S) is the expected value, R(n) is the range of the first n values, S(n) in their standard deviation, n is the number of data points and c is a constant.

## 4. Description of Data Experimental Time Series

We analyzed below one of the most unusual ICME and SEP events that were detected by STEREO A and B in November 2007. According to the STEREO ICME list (see event 6 from http://www-ssc.igpp.ucla.edu/forms/stereo/stereo_level_3.html), a quite typical magnetic cloud or flux rope passed through the STEREO A position from 22:00 UT, 2007 November 19 to 3:17 UT, 2007 November 21. The most intriguing fact about the ICME was that energetic ion flux enhancements were detected not where they were supposed to be (a bit ahead of the ICME shock and within the ICME). Instead of that, substantial energetic particle enhancements were observed in areas where SEPs are not usually expected, namely, prior to and after the magnetic cloud passage. Therefore, the picture of particle acceleration was unusual.

**Figure 1:**
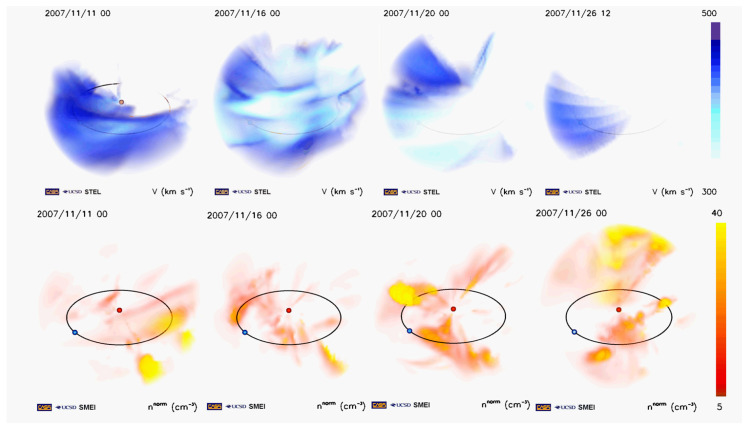
Velocity (**upper panel**) and density (**lower panel**) 3-D reconstructions before, after and during the passage of an Interplanetary Corona Mass Ejections (ICME) surrounded by the rippled Heliospheric current sheet, which led to the occurrence of regions filled with trapped energetic particles around the ICME (modified from [1]). All measurements are recalculated to the Earth fixed position (the blue dot). The heliographic longitude of STEREO A was 20.220 deg (in the anticlockwise direction with respect to the Earth) and the heliolatitude was –0.311 deg. (Exact coordinates of the STEREO A location for the specified period can be found, for example, at https://stereo-ssc.nascom.nasa.gov/cgi-bin/make_where_gif).

The findings mentioned above, were presented and discussed by Khabarova et al. [1] in terms of understanding the large-scale topology of streams and showing the occurrence of SMIs in ripples of the HCS surrounding an ICME. It was noted that the regular structure of the HCS was significantly distorted by pre-existing multiple ICMEs and co-existing long-lived coronal hole flows from 16 to 26 November 2007. As a result, the HCS possessed a non-planar form of rose-leaves and was full of numerous ripples during the period of propagation of the ICME (see Figure 1). Such ripples and sandwich-like current sheets filled with numerous small-scale magnetic islands represent magnetic traps or so-called magnetic cavities for energetic particles of keV-MeV energies. Therefore, energized particles accelerated during a flare and/or the propagating ICME driven-shock were subsequently confined by the HCS, possessing a complicated structure, which prevented them from streaming away freely.

This scenario can be illustrated by 3-D reconstructions of the SW density obtained from white light measurements of the Solar Mass Ejection Imager (SMEI), and Solar Terrestrial Environment Laboratory (STEL) velocity reconstructions, which are derived from the interplanetary scintillation data http://smei.ucsd.edu/new_smei/data&images/data&images.html [70,71,72,73]. SW velocity profiles approximately correspond to Interplanetary Magnetic Field (IMF) spatial variations [74], the spatial distribution of the IMF cannot be reconstructed, looking at the upper blue panel in Figure 1. The corresponding density profiles are shown in the lower red panel.

STEL measurements show that the initially quiet HCS (2007/11/11) was strongly disrupted by multiple propagating and co-existing ICMEs seen as numerous red peculiarities in the density picture on 2007/11/16. Coronal hole flows are seen as extended yellow regions here, and the corresponding upper figure shows a rose-leaf structure of the HCS. Those HCS leaves and ripples surrounded the ICME detected by STEREO A on 2007 November 19. The ICME is seen as an extended region near the Earth deflected from a cone-like coronal hole to the right from the Earth in the density picture on 2007/11/20. There are two other merged ICMEs with denser leading edges seen in the same picture at higher latitudes, which did not strike the Earth. The HCS structure could not get a usual planar form during this period either, according to STEL observations (see the upper panel). Finally, it obtained some balance after the passage of the ICME, but still STEL reveals the rippled form of the HCS. Small-scale magnetic islands were detected within the HCS ripples, as shown with the IMF vector rotation holograms in [1]. 

**Figure 2:**
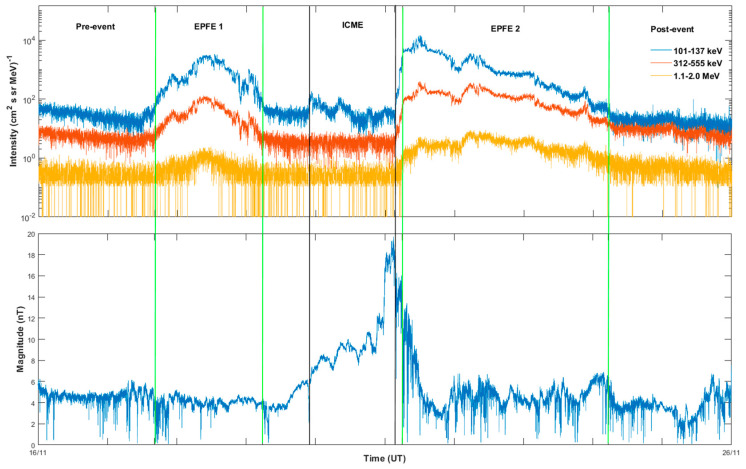
Energetic particle flux enhancements (EPFEs), observed by the STERO A spacecraft, caused by the ICME interaction with the rippled HCS: (**upper panel**) Energetic ion flux in the three energy channels from 101 keV to 2 MeV. EPFEs are not observed during the ICME magnetic cloud passage (bounded by black vertical lines), but there are two strong increases associated with the areas filled with magnetic islands (areas bounded by green vertical lines); (**bottom panel**) Interplanetary Magnetic Field (IMF) magnitude in Radial Tangential Normal (RTN) coordinates for the same period.

Figure 2 shows the behavior of the energetic particle flux and the IMF strength for the period from 00:00 UT, 16 November to 00:00 UT, 26 November 2007 as observed by the STEREO A spacecraft near 1 AU. The data were obtained directly by the SEPServer data server (http://server.sepserver.eu/index.php) for energetic particle flux. Figure 2 (upper panel) shows the energetic ion flux variations in the three energy ranges: 101–137 keV, 312,555 keV, and 1.1–2.0 MeV from the STEREO A/IMPACT/SEPT experiment [74]. Figure 2 (bottom panel) presents the IMF observations from the STEREO A/IMPACT Magnetic Field Experiment [75] during the same period. The data were obtained directly by the UCLA data server (http://aten.igpp.ucla.edu/forms/stereo/heliocentric_level1_magnetic_field.html). The period between the two black vertical lines indicates the passage of the ICME according to the ICME catalogue based on characteristic signatures of the magnetic cloud passage (http://www-ssc.igpp.ucla.edu/~jlan/STEREO/Level3/STEREO_Level3_ICME.pdf). The periods delineated as “EPFE1” and “EPFE2” in Figure 2, are the two periods with strong particle increases, associated with the areas filled with magnetic islands. The boundaries of these periods have already been identified in the work of Khabarova et al. [1] and indicated by the yellow stripes in their Figure 3. The precise selection of these periods was done based on the time the elevated level-decreasing level of the energetic particle flux starts to be observed. Khabarova et al. [1] showed that the two strong energetic particle flux enhancements (i.e., “EPFE 1” & “EPFE 2”) bounded by the green vertical lines in Figure 2, are associated with areas filled with SMIs trapping and re-accelerating SEPs and energetic particles accelerated at the ICME shock, as noted above.

**Figure 3:**
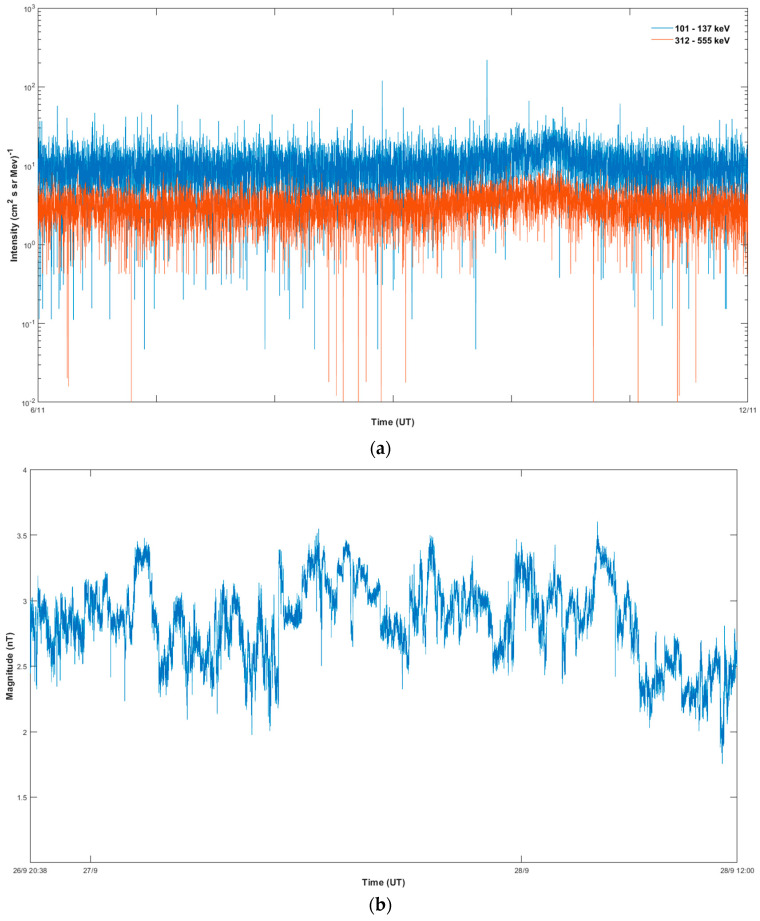
Quiet period observed during the identified “quiet” periods for: (**a**) Energetic ion intensities; (**b**) magnetic field.

In order to carry out the statistical analysis suggested above, we have identified several periods characterizing abrupt changes in the observed variations of the energetic ion flux intensities. First, we identified a “quiet” period in terms of the energetic ion flux behavior for the comparison with periods of the interest. We selected this specific interval based on the intensity of energetic ions being in the background level. The “quiet” period is shown in Figure 3, it lasts from 00:00 UT, 6 November 2007 till 00:00, 12 November 2007. We display the observed energetic ion flux for the period separately in Figure 3a to demonstrate the background level of intensities in the corresponding energy channels. Similarly, we identified a “quiet” period in terms of magnetic field magnitude behavior for the comparison with periods of the interest (Figure 3b) and it lasts from 20:38 UT, 26 September 2007 till 12:00 UT, 28 September 2007. Second, we selected a “pre-event” period from 00:00 UT, 16 November 2007 till 16:21 UT, 17 November 2007. We chose this interval between a previous unrelated particle enhancement (not shown) and the start of the “EPFE 1” period. The period between 16:22 UT, 17 November and 05:45, 19 November 2007 corresponds to “EPFE 1”, denoted by green vertical lines in Figure 2. The “ICME” magnetic cloud passage covers the period from 22:00 UT, November 19 to 3:17 UT, 21 November 2007. The “EPFE 2” period lasts from 06:00 UT, 21 November 2007 till 05:16 UT, 24 November 2007. Finally, the “post-event” period was identified from 05:17 UT, 24 November to 00:00 UT, 26 November 2007. This interval was chosen at the end of the “EPFE 2” period till the time when the energetic ion intensities are observed to return to their background level (not shown).

In Figure 2, there is a strong difference in the behavior of the energetic ion intensities observed in periods EPFE 1 and EPFE 2, with respect to the magnitude and the time duration. Below, we relate these differences with the stronger multifractality and non-extensivity of the plasma system as it is shown by the Tsallis q-triplet and the multifractal parameters of the plasma dynamics. The energetic particle flux enhancements can be explained by the existence of magnetic islands, which create intermittent magnetic turbulence as Khabarova et al. [1] has shown to occur during EPFE 1 and EPFE 2 periods. As we explain in the discussion section, the magnetic island associated dynamical processes can produce the electric field induced by intermittent turbulence, suggesting the occurrence of evolving structures and anomalous diffusion with simultaneous fractional–anomalous acceleration processes.

## 5. Data Analysis and Results

The analysis has been carried out for TMS of the energetic ion intensities observed in the 312–555 keV energy range. Similar results (not shown) were obtained for the energetic ion intensities of 101–137 keV energies.

### 5.1. Flatness Coefficient Estimation

**Figure 4:**
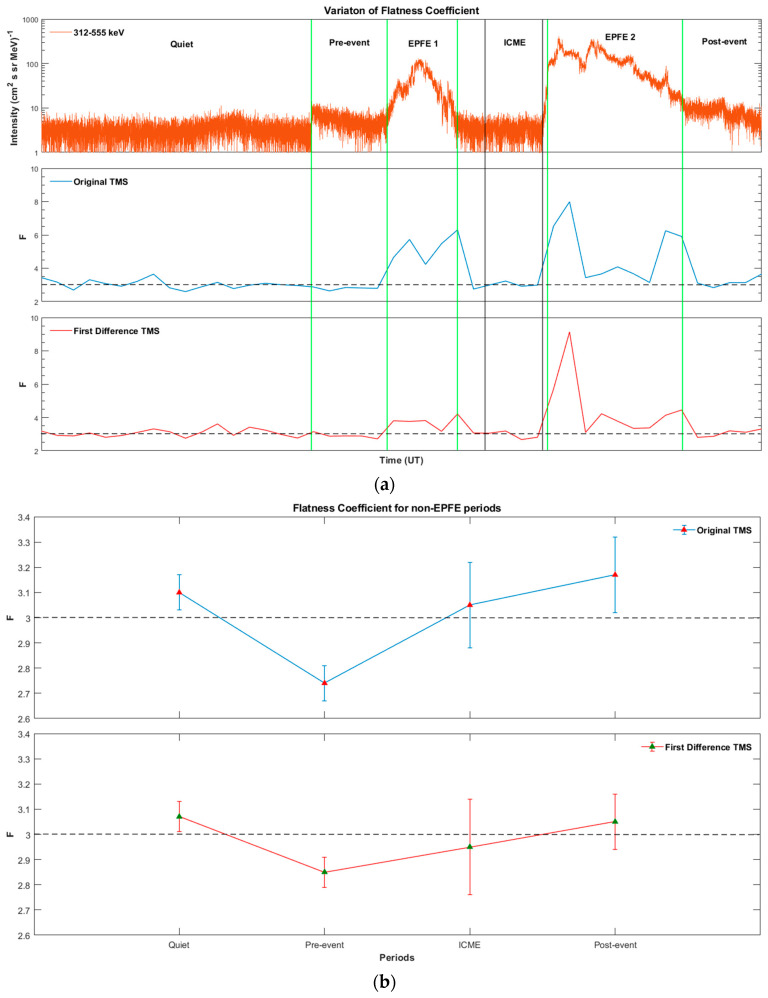
Variation of the Flatness coefficient of energetic particle fluxes during the event. The blue line corresponds to original Time Series (TMS), while the red line corresponds to first difference TMS. The dashed lines indicate the “limit” of Gaussian character of the system: (**a**) (**upper panel**) Energetic ion fluxes in 312–555 keV energy range, (**middle panel**) Flatness coefficient of original TMS, (**bottom panel**) Flatness coefficient of first difference TMS; (**b**) mean value of Flatness coefficient for periods quiet, pre-event, ICME, and post-event. (**Upper panel**) original TMS, (**bottom panel**) first difference TMS.

**Figure 5:**
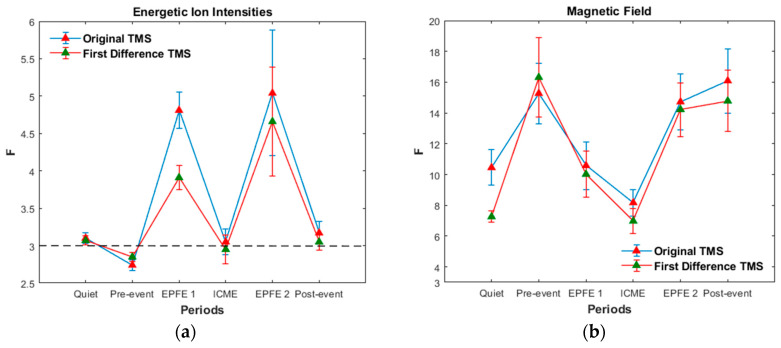
Mean value of Flatness coefficient for each period. The blue lines correspond to original TMS, while the red lines correspond to first difference TMS. The dashed lines indicate the “limit” of Gaussian character of the system: (**a**) Energetic ion intensity TMS; (**b**) Magnetic field TMS.

Figure 4 presents the variation of Flatness coefficient *F* of energetic particle fluxes, during the whole period under study. Figure 5 presents the mean value of Flatness coefficient for each period, for the original energetic ion intensity and magnetic field TMS, as well as for the first differences of the original TMS, correspondingly. The first difference TMS describe processes in smaller scales in comparison with the original signals. As concerns the energetic particles, during EPFE periods corresponding to the strong enhancements of energetic particle fluxes, the Flatness coefficient is clearly much higher than the value of 3 (Figure 4a and Figure 5a). For the quiet, pre-event and post-event periods, the Flatness coefficient fluctuates near the value of 3 but remains different from 3, while for the ICME period the mean value of Flatness coefficient is different from 3 but the error bars include the value of 3 (Figure 4b).

In order to decide about the non-Gaussian character of the non-EPFE periods, we used the quantile-quantile (q-q) plot (not shown here), which showed deviation from linearity (Gaussianity). Moreover, we used the Anderson and Darling statistical test [76], one of the most powerful statistical tool for detecting deviation from normality (Gaussianity). The results of this test for quiet, pre-event, ICME and post-event periods reject the null hypothesis, which means a clear deviation from the Gaussian (linear) character of the statistics. The non-Gaussian character of the energetic particle signals in quiet, pre-event, ICME, and post-event periods, is also supported by the results of the other diagnostic tools presented in the following paragraphs.

After all, it is clear that the system passes from near Gaussian (weak non-Gaussian) state to a strong non-Gaussian state, both for original and first difference TMS (Figure 5a). In this sense, the terms “near Gaussian–weak non-Gaussian” corresponds to a clearly non-Gaussian process. As concerns the magnetic field measurements, the estimation of the mean value of Flatness coefficient for each period, indicates a strong non-Gaussian character for all the periods (Figure 5b).

Table 1 and Table 2, present the mean value of the Flatness coefficient for the energetic ion intensity and magnetic field TMS, respectively.

In the following we present results concerning the magnetic field, as we have significant theoretical reasons to believe the existence of physical connection of the magnetic field and the energetic particle profiles (see Appendix A.1.1–Appendix A.1.3). In the magnetic field TMS (original and first difference), the non-Gaussian character is presented during all periods as the coefficient *F* takes values ≫3, much higher than the Gaussian value (*F* = 3). In particular, the enhancement of non-Gaussian character is observed for pre-event, EPFE 2, and post-event periods, as the Flatness coefficient during these periods increases to much higher values than the other periods (quiet, EPFE 1, ICME). It is noticeable that during the pre-event period there is a strong enhancement of the non-Gaussian character of the magnetic field. According to our theoretical framework (see Appendix A), the enhancement of magnetic field non-Gaussian character must be generally related someway to the energetic particles enhancement. However, this concept must be further testified by using rich statistics of SMIs events.

### 5.2. Tsallis q-Triplet Estimation

(a) Tsallis $q_{sen}$

**Figure 6:**
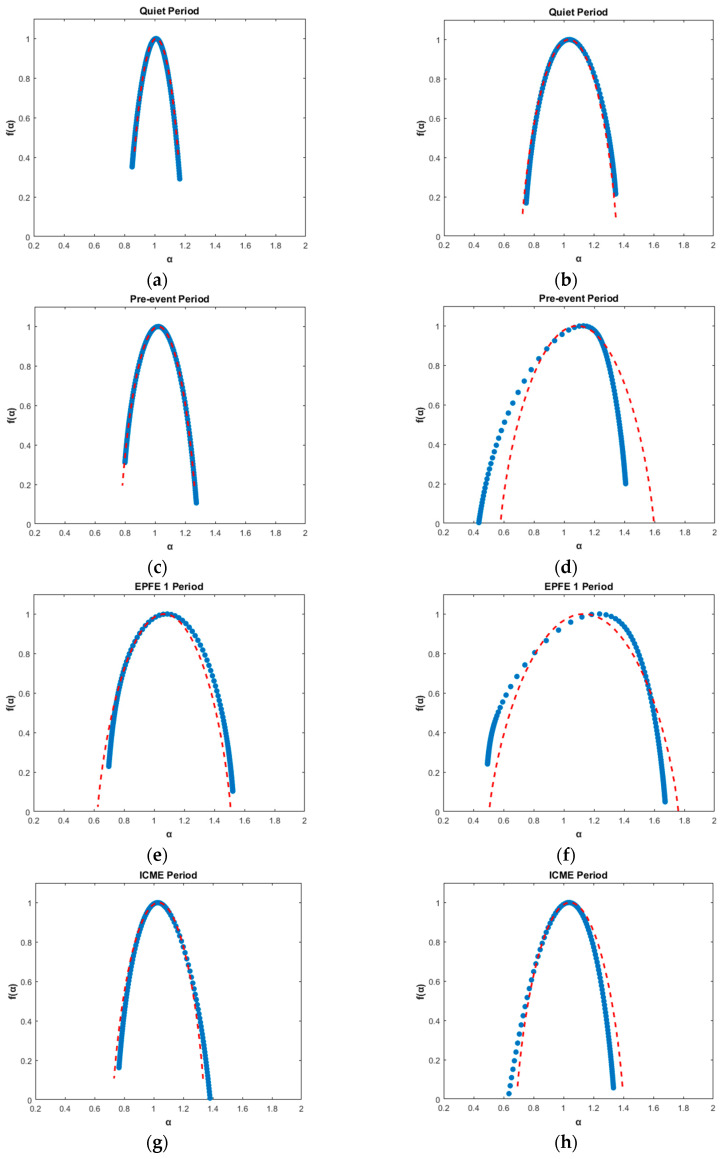

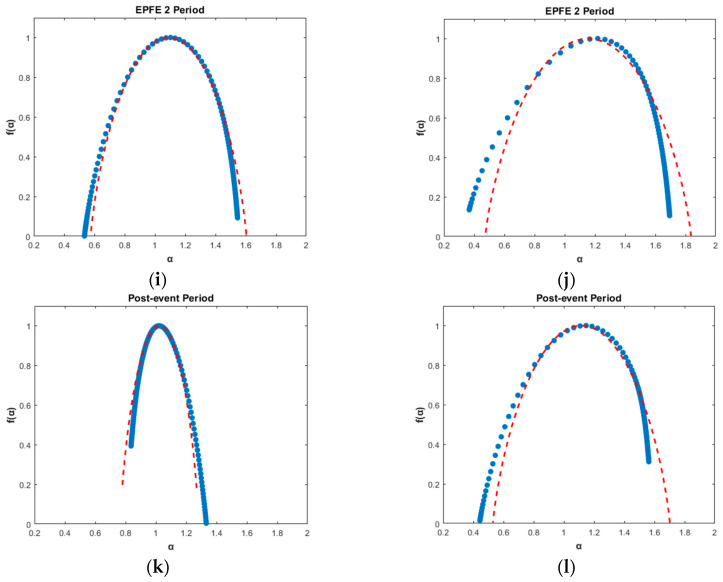
Singularity spectrum for energetic ion intensity (**left column**) and magnetic field (**right column**) TMS, shows the related singularity spectra f(α) as a function of singularity strength α. The red dashed line corresponds to a nonlinear regression best fit of the data (p-model): (**a,b**) Quiet periods; (**c,d**) pre-event periods; (**e,f**) EPFE 1 periods; (**g,h**) ICME periods; (**i,j**) EPFE 2 periods; (**k,l**) post-event periods.

Figure 6 shows the related singularity spectrum f(α) as a function of singularity strength α for energetic ion intensity, Figure 6 (left column), and magnetic field, Figure 6 (right column), TMS, corresponding to six periods (quiet, pre-event, EPFE 1, ICME, EPFE 2, and post-event) as captured from the STEREO A spacecraft. The red dashed line is a nonlinear regression best fit of the experimental data making use of an analytical expression for tow-scale Cantor set with equal scales but unequal weights (p-model). As one can see in Figure 6, the multifractal character changes as we pass from the quiet to the next periods and becomes stronger during the EPFE periods. However, there are significant differences between the multifractal curves which can be quantified using the Tsallis $q_{sen}$ index, as well as other geometrical characteristics of the singularity spectrums such as the value $\alpha_{0}$, the degree of multifractality Δα, the degree of asymmetry *A*, and the generalized dimension spectrum $D_{\bar{q}}$ [77,78].

First, we present results concerning parameters describing the geometrical properties of the curve of singularity spectrum f(α). Comparing the values of the four parameters obtained from the singularities spectrum, we observe the followings: The parameter $\alpha_{0}$ for EPFE 1 and EPFE 2 periods was found to be higher than the other periods (Table 3). Similar results obtained for the magnetic field TMS (Table 4). Therefore, $\alpha_{0}$ is smaller for the f(α) spectrum of quiet, ICME, pre-event and post-event periods, in both cases of energetic ion intensity and magnetic field TMS. Smaller values of $\alpha_{0}$ indicate underlying processes with rather regular appearance (e.g., smoother TMS) since $\alpha_{0}$ is an indicator of the strength of the largest fluctuations [79]. Therefore, our estimation of $\alpha_{0}$ for the analyzed event denotes that the energetic ion flux variations observed during the quiet, ICME, pre-event, and post-event periods are more regular (e.g., less erratic) than the corresponding ones occurring during the EPFE 1 and EPFE 2 periods.

Moreover, the estimation of the degree of multifractality Δα showed that Δα is greater during the EPFE 1 and EPFE 2 periods. Thus, higher values of Δα indicate that the underlying process is richer in fractal terms, meaning that both the energetic particle ions and magnetic field exhibits more complex multifractal properties during the EPFE periods, than the corresponding quiet, ICME, pre-event, and post-event periods.

The degree of asymmetry *A* essentially gives information on the asymmetry of the singularity spectrum. When *A* = 1, f(α) is symmetric. For the energetic particle ions, the analysis reveals that the singularity spectrum of the pre-event period is the most symmetric since, for this period, the parameter was found to be A=1.006±0.084≈1. The results reflect the fact that the spectrum is almost non-skewed (A≈1) for the quiet, pre-event and EPFE 1 periods, left-skewed *A* > 1 for the EPFE 2 period, while for the ICME, and post-event periods it is right-skewed, since *A* < 1. Therefore, for the period during which *A* > 1 (EPFE 2) the spectrum is left-skewed, indicating a dominance of small fractal exponents α related to an abundance of small fluctuations in the TMS, while for the periods during which *A* < 1 (ICME and post-event periods) the spectrum is right-skewed, indicating a dominance of large fractal exponents α related to an abundance of large fluctuations in the TMS. For the case when A≈1 (quiet, pre-event, and EPFE 1 periods) the low and high fractal exponents α, are of equal importance meaning an equilibrium of small and high fluctuations in the TMS.

Similarly, for the magnetic field the analysis reveals that the singularity spectrum of the quiet period is most symmetric since the parameter was found to be A=0.935±0.006≈1. In this case, as one can see, for all periods except the quiet period, the results reflect the fact that the spectrum is left-skewed (*A* > 1), indicating a dominance of small fractal exponents α related to an abundance of small fluctuations in the TMS.

The results of the singularity spectrum presented in Table 3 reveal significant differences between the singularity curves, indicating also differences between the corresponding processes that cause temporal variations of the energetic ion intensities. These obtained results are also verified by the estimation of Tsallis $q_{sen}$ index for the energetic particle ions, which revealed $q_{quiet}<q_{pre-event}≅q_{post-event}<q_{ICME}<q_{EPFE1}<q_{EPFE2}$. Similarly for the magnetic field, the obtained results for the singularity spectrum (Table 4) are also verified by the estimation of the $q_{sen}$ index, which revealed $q_{quiet}<q_{ICME}<q_{pre-event}<q_{post-event}<q_{EPFE1}<q_{EPFE2}$.

In Table 3 and Table 4, we summarize all the parameters $\alpha_{0}$, *A*, Δα, $\Delta D_{\bar{q}}$, p-model, and $q_{sen}$, with regard to the multifractal profile of the particle intensities and distributions, as well as of the magnetic field, as they were calculated in the SW plasma for all periods. The energetic multifractal profile of these periods described by the characteristic values of the multifractal parameters are included in Table 3 and Table 4. As one can see, all of the parameters during EPFE 1 and EPFE 2 periods simultaneously increase, for both the energetic ion intensity and magnetic field TMS. Moreover, we obtain that the parameters during period EPFE 2 are higher than the period EPFE 1, in both cases.

According to Sen [79], in the case when $q_{sen}<1$, this yields a power law behavior (instead of exponential, which indicates BG-statistics) for the sensitivity of initial conditions: $\xi \propto t^{\lambda_{qsen}}=t^{1/\left(1-q_{sen}\right)},\;\left(t\to \infty \right)$, where ξ is the distance of neighboring trajectories. Therefore, in both cases of energetic ion intensity and magnetic field TMS, according to the values of the Tsallis $q_{sen}$ and using the q-generalized Pesin-like identity ($K_{q_{ent}}\equiv \lambda_{q_{sen}}$ with $q_{ent}=q_{sen}$), the processes related to the EPFE 1 and EPFE 2 periods are connected with a greater loss of information in phase space, as $\xi_{quiet}=t^{0.396}<\xi_{pre-event}=t^{0.562}≅\xi_{post-event}=t^{0.566}<\xi_{ICME}=t^{0.655}<\xi_{EPFE1}=t^{0.962}<\xi_{EPFE2}=t^{1.248}$, and greater rate of entropy production as $K_{quiet}<K_{pre-event}≅K_{post-event}<K_{ICME}<K_{EPFE1}<K_{EPFE2}$. Similarly, for the magnetic field TMS, the loss of information in phase space is: $\xi_{quiet}=t^{0.693}<\xi_{ICME}=t^{1.215}≅\xi_{post-event}=t^{1.605}<\xi_{post-event}=t^{1.718}<\xi_{EPFE1}=t^{1.848}<\xi_{EPFE2}=t^{2.653}$, and the rate of entropy production is: $K_{quiet}<K_{ICME}<K_{pre-event}<K_{post-event}<K_{EPFE1}<K_{EPFE2}$.

**Figure 7:**
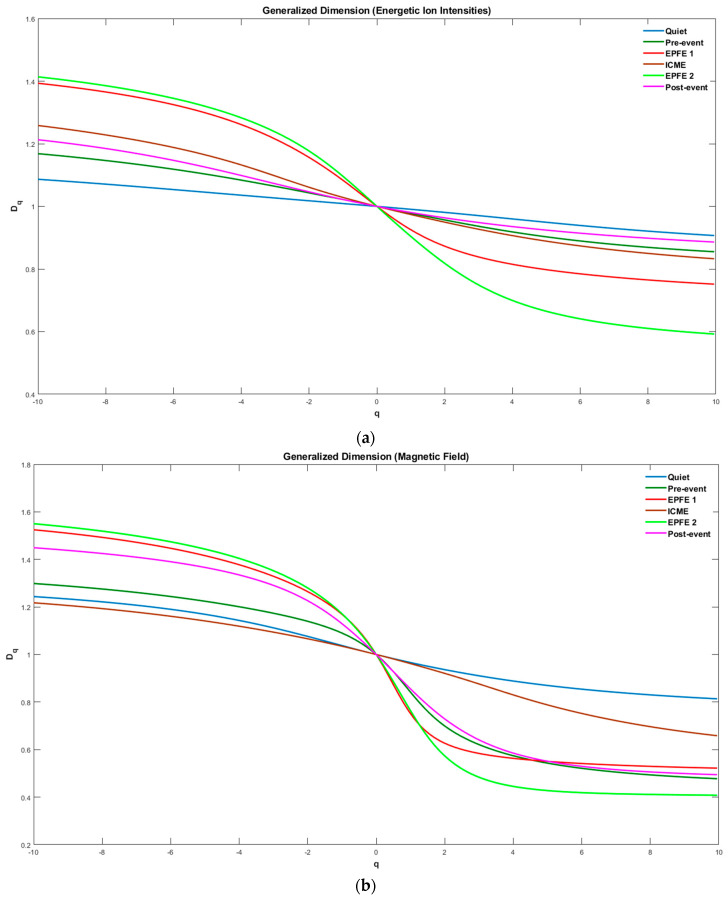
Generalized dimension spectrum for all examined periods: (**a**) Energetic ion intensity TMS; (**b**) Magnetic field TMS. The blue line corresponds to quiet period, the dark green line to pre-event period, the red line to EPFE 1 period, the brown line to ICME period, the light green line to EPFE 2 period and the magenta line corresponds to post-event period.

The singularity spectrum f(α) is related to the generalized dimension spectrum $D_{\bar{q}}$, as described above (see Equation (5)). The generalized spectrum $D_{\bar{q}}$ for positive values of $\bar{q}$, describes low dimensional regions in the phase space, while the negative values of $\bar{q}$ describe high dimensional regions in the phase space. Figure 7 shows, the generalized dimension spectrum $D_{\bar{q}}$, for all periods during which the f(α) spectrum was estimated. For periods 3 (EPFE 1) and 5 (EPFE 2), one can see a strong difference of $\Delta D_{\bar{q}}$ between the low $\left(\bar{q}\to +\infty \right)$ and high $\left(\bar{q}\to -\infty \right)$ dimensional regions of the phase space of space plasma dynamics, and the values of $\Delta D_{\bar{q}}$. The generalized dimension for $\bar{q}=2$ corresponds to Correlation Dimension [64]. As one can see, the generalized dimensions for $\bar{q}=2$ is lower in EPFE 2 period than the EPFE 1 period, for both energetic ion intensity (Figure 7a) and magnetic field (Figure 7b) TMS. In Figure 7a, the value of $\Delta D_{\bar{q}}$ for EPFE 1 and EPFE 2 periods was found to be higher than 0.6. For the rest of the periods, the difference $\Delta D_{\bar{q}}$ remains smaller than 0.35, except for period 4 (ICME), during which $\Delta D_{\bar{q}}$ was found to be between 0.35 and 0.45. Moreover, it can be observed that the difference $\Delta D_{\bar{q}}$ is strengthened during period 5 (EPFE 2) (green curve) to the value 0.822 compared with the value of 0.641 estimated for the period 3 (EPFE 1) (red curve). The strong difference in the multifractal character seen during periods 3 (EPFE 1) and 5 (EPFE 2), which is reflected in the $\Delta D_{\bar{q}}$ and Δα values, is related with the physical mechanism of energetic particle production. According to the obtained results, the mechanism can be described as fractional acceleration process, as the multifractal character of phase space is strengthened. Moreover, the skewness is impacted by the temporal profiles observed during the EPFEs periods themselves.

Certainly, the topology of an event and the location of the spacecraft with respect to it may impact the characteristics of energetic particles and, consequently, the results of the statistical analysis. It is noteworthy that observations have shown that EPFEs (or AEPEs) observed in low-energy channels (up to 5 MeV) have a local origin. Such features as the detection of specific variations in the energetic ion flux along with propagation of the SW as detected by different spacecraft and the specific non-smooth profile of the energetic ion flux sitting on top of pre-existing SEP events and correlated with crossings of magnetic islands provide evidence in favour of that supposition. In this particular case, the restored IMF topology suggests formation of numerous magnetic cavities in HCS ripples. The ICME was very unusual in all senses and impacted by pre-existing streams. It is very unusual to see EPFEs well before and after the passage of an ICME. Especially the latter, taking into account that the solar source does not operate anymore, and there is no shock at the ICME trailing edge strong enough to produce an individual hump of the energetic particle flux peaking far from it. All together these points suggest that an additional mechanism of particle acceleration exists. Interplanetary Scintillation (IPS) and ENLIL (http://helioweather.net/) reconstructions show that in similar cases the trailing edge of the ICME is highly skewed and forms a magnetic cavity confined more effectively than the pre-ICME one. As a result, magnetic reconnection producing magnetic islands occurs with an increased rate. Even visual inspection shows an increased number of drops in |B|, corresponding to crossings of current sheets separating magnetic islands, in the second EPFE period. One can suggest that the obtained statistical properties in regions EPFE1 and EPFE2 are mostly determined by the physical properties of the magnetic cavities containing magnetic islands, which, in turn, trap and re-accelerate energetic particles.

Furthermore, according to Zank et al. [45,46,47], le Roux et al. [48,49,50,51], Adhikari et al. [2], the effectiveness of the acceleration strongly depends on the characteristics of magnetic islands and magnetic cavities, confining them, which is different in EPFE1 and EPFE2 periods as already highlighted above. It is quite obvious that since the effect of local particle acceleration is stochastic and collective, the number of magnetic islands and the occurrence of larger-size magnetic islands change the efficiency of the acceleration considerably. In this term, the larger number of magnetic islands in the EPFE2 period can well explain the observations and this is what is meant by the impact of the topology on the characteristics of energetic particles and, consequently, the results of the statistical analysis.

It is noteworthy that the EPFEs were not observed only by single-point measurements. The unusual double enhancements were detected by STEREO-A and STEREO-B with a corresponding time shift, as it can be checked at http://www2.physik.uni-kiel.de/stereo/browseplots/index.php. The separation angle between the two spacecraft was ~40 deg. L1 spacecraft also detected the event. The separation angle with the Earth is ~20 deg. Particle acceleration in magnetic islands occurs very quickly (see [45]). Therefore, there is a strong reason to consider that we are dealing with spatial changes, observing a corresponding propagation of the magnetic cavities.

In Figure 7b for the magnetic TMS, we observe similar results with energetic ion intensities (Figure 7a). Once again, for EPFE 1 and EPFE 2 periods, one can see a strong difference of $\Delta D_{\bar{q}}$ between the low $\left(\bar{q}\to +\infty \right)$ and high $\left(\bar{q}\to -\infty \right)$ dimensional regions of the phase space of space plasma dynamics. The values of $\Delta D_{\bar{q}}$ were found to be larger than 1 for these periods, while for the rest of the periods, the difference $\Delta D_{\bar{q}}$ remains smaller than 1. It can be observed that the difference $\Delta D_{\bar{q}}$ is strengthened during EPFE 2 (green curve) than the EPFE 1 period (red curve).

During periods 3 (EPFE 1) and 5 (EPFE 2), the energetic particle intensities rise to values above $10^{2}\;ions{\left(cm^{2}\cdot sr\cdot MeV\right)}^{-1}$ (see Figure 2), in contrast with a quiet time background of the energetic particle intensities which is between $10^{0}-10^{1}\;ions{\left(cm^{2}\cdot sr\cdot MeV\right)}^{-1}$ as seen in Figure 3. At the same time, the multifractal profile of the energetic particle distribution is strengthened as the indices Δα, $\Delta D_{\bar{q}}$ and $q_{sen}$ show clearly enhanced profiles. This is strong evidence for fractional acceleration processes as the phase space of the system dynamics reveals a strong topological phase transition from a low to a high multifractal profile, since the multifractal parameters Δα, $\Delta D_{\bar{q}}$ and $q_{sen}$ reach much higher values than during quiet periods. Comparing the degree of asymmetry *A*, between EPFE 1 and EPFE 2 periods, we observe that *A* < 1 in the first period, while *A* increases to value higher than 1 in the second one. This enhancement of the asymmetry index *A* from period 3 (EPFE 1) to the period 5 (EPFE 2), is also associated with higher values of the energetic particle flux. Utilizing the observed intensities of the energetic particles, we calculated the total number of ions detected by the spacecraft during each EPFE period (i.e., the particle fluence). It was found that the EPFE 1 period comprised a particle fluence of $1.75\times 10^{6}\;ions{\left(cm^{2}\cdot sr\cdot MeV\right)}^{-1}$, while a much higher particle fluence of $10.5\times 10^{7}\;ions{\left(cm^{2}\cdot sr\cdot MeV\right)}^{-1}$ was estimated during EPFE 2. The difference in the energetic particle fluence values is caused by the higher intensity values and the longer duration time of the EPFE 2 period, compared with the EPFE 1 period. We know that the asymmetry index *A* depends upon the extension of low and high dimensional regions in phase space. As we have described previously (description of Equation (7)), when *A* is larger than one the density of low dimensional regions described by the right part of the generalized dimension spectrum $D_{\bar{q}}$, is higher than the density of high dimensional regions of the phase space described by the left values of $D_{\bar{q}}$. Thus, in the case of *A* > 1 corresponding to EPFE 2 period the mechanism of fractional acceleration process is strengthened, producing more energetic particle populations. This theoretical explanation of observations is in agreement with the profile of the generalized dimension spectrum shown in Figure 7a, where the estimated low dimensional part of $D_{\bar{q}}$ spectrum $\left(\bar{q}>0\right)$ for the EPFE 2 period (green curve), is lower than the corresponding low dimensional part of $D_{\bar{q}}$, estimated for the EPFE 1 period (red curve). We must note here, that the low dimensional regions of the phase space are associated with weak fluctuations and strong singularity of the topology of the phase space. This strong anomalous profile also produces strong anomalous diffusion and strong fractal acceleration processes. On the contrary, the high dimensional regions of phase space are related with large fluctuations and smoother topology of the phase space. As a result, the fractional acceleration process is weaker during the period 3 (EPFE 1), as it is concluded comparing the physical non-extensive states of the system at the energetic flux enhancements observed in period 3 (EPFE 1) and period 5 (EPFE 2), as shown in Figure 7a. Also, the multifractality and intermittent turbulence profile of magnetic field indicated by parameters Δα, $\Delta D_{\bar{q}}$, $q_{sen}$, become stronger for EPFE periods and even stronger in EPFE 2 than in EPFE 1. The above results concerning energetic particles and magnetic field, showing the similar change of multifractality and intermittency, reveals the physical connection of the magnetic field underlying dynamics and the underlying dynamics of the energetic particles acceleration.

**Figure 8:**
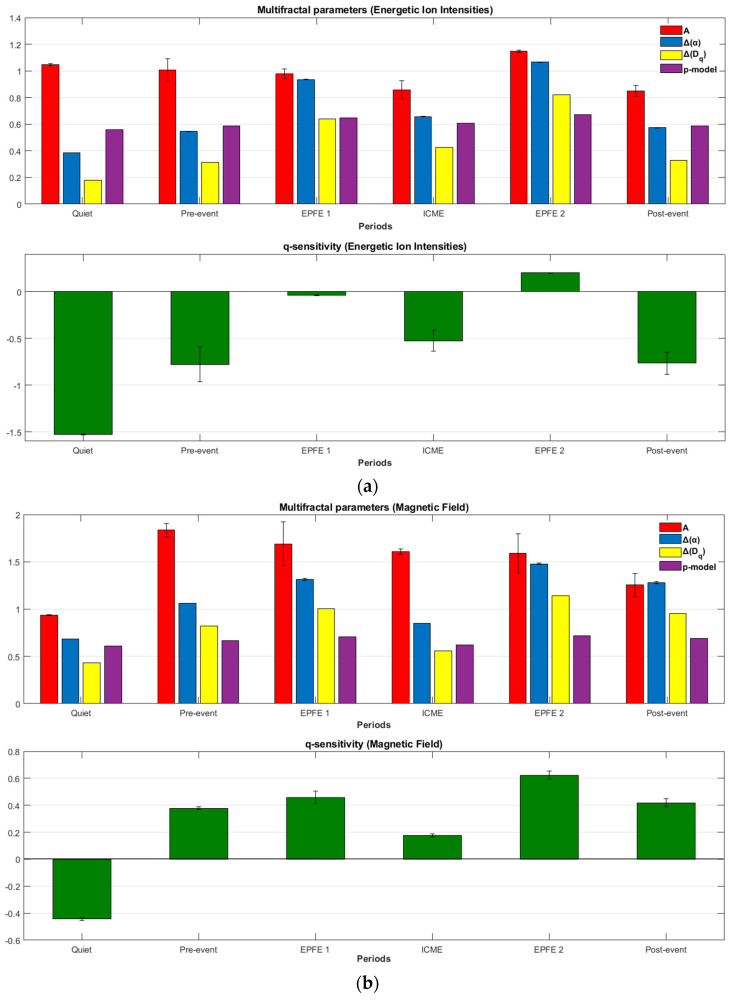
Characteristics parameters of singularity spectrum profile for all periods under study: (**a**) Energetic ion intensity TMS; (**b**) magnetic field TMS.

In Figure 8, we visually illustrated the results from Table 3 and Table 4. As one can see in the upper panels of Figure 8a,b, there is observed simultaneous increases of $\Delta D_{\bar{q}}$ (yellow bar), Δα (blue bar), and p-model (magenta bar) in the EPFE periods, for both energetic ion intensity (Figure 8a) and magnetic field (Figure 8b) TMS. Also, there is a strong enhancement of the $q_{sen}$ index (green bar) for the same periods. Moreover, the above parameters are higher in the EPFE 2 period than the period EPFE 1, for both TMS.

(b) Tsallis $q_{rel}$

**Figure 9:**
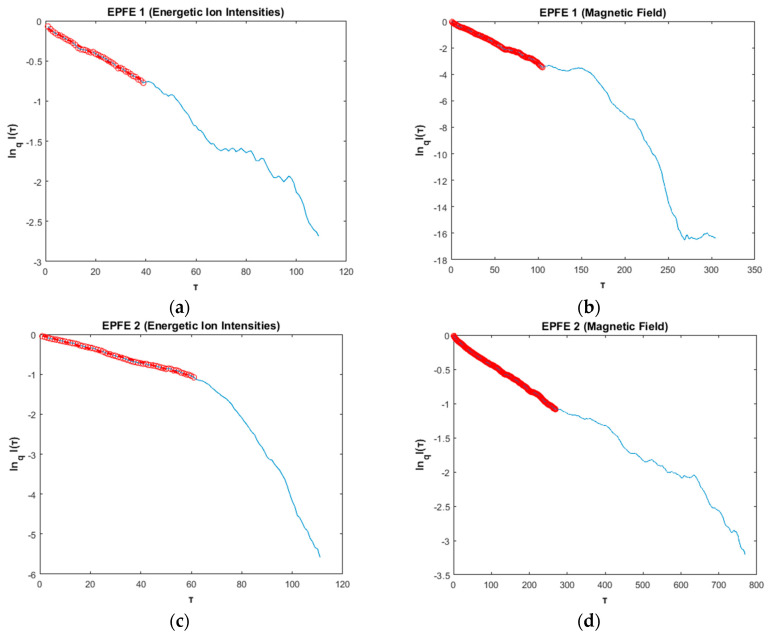
The linear fit on mutual information curve: (**a**) EPFE 1 period for energetic ion intensities; (**b**) EPFE 1 period for magnetic field; (**c**) EPFE 2 period for energetic ion intensities; (**d**) EPFE 2 period for magnetic field.

In Figure 9 we present the best $\ln_{q}I\left(\tau \right)vs\tau$ fitting of the mutual information function for the EPFE periods of energetic ion intensity (left column) and magnetic field (right column) TMS. With the red circles, we emphasize the linear fit used for the estimation of $q_{rel}$ index, according to Equation (12). For the energetic ion intensities, the results showed that the $q_{rel}$ index was found to be $q_{rel}=7.43\pm 0.14$ for the EPFE 1 period (Figure 9a), and for the EPFE 2 period the $q_{rel}$ index was found to be $q_{rel}=8.63\pm 0.18$ (Figure 9c). For the rest of the periods (quiet, pre-event, ICME, post-event), the estimated $q_{rel}$ values, are found to fluctuate near the value 1 as the relaxation time is very quick. After this, for the EPFE periods, the Tsallis parameter $q_{rel}$ remains different than unity and this reveals a non-Gaussian relaxation process of the system to its NESS, while for the rest of the periods the Tsallis parameter $q_{rel}$ remains close to unity and this reveals a near-Gaussian relaxation process of the system to its NESS. Similarly, for the magnetic field, the results showed that the $q_{rel}$ index was found to be $q_{rel}=4.25\pm 0.06$ for the EPFE 1 period (Figure 9b), whereas for the EPFE 2 period the $q_{rel}$ index was found to be $q_{rel}=5.65\pm 0.09$ (Figure 9d). 

In addition, for the EPFE periods Tsallis q_rel index was found larger than the other periods, a result that indicates a slow relaxation process approach to NESS. The quiet, ICME, and pre/post event periods are characterized by very fast relaxation process. These results indicate strong self-organization of the SW plasma system during the EPFE periods and caused by some kind of topological phase transition process of the system dynamics. As we show in the following, the q_rel parameter increases as the non-extensivity and the multifractality of the system becomes stronger. In other words, as the q_sen and q_stat parameters are increasing, the q_rel parameter increases too. The slow relaxation of the system at the energetic flux enhancement periods (EPFE1 and EPFE2), is caused by the holistic behavior of the system as the self-organization (reduction of dimensionality) process is strengthened at the states with strong multifractality and non-extensive character.

(c) Tsallis $q_{stat}$

**Figure 10:**
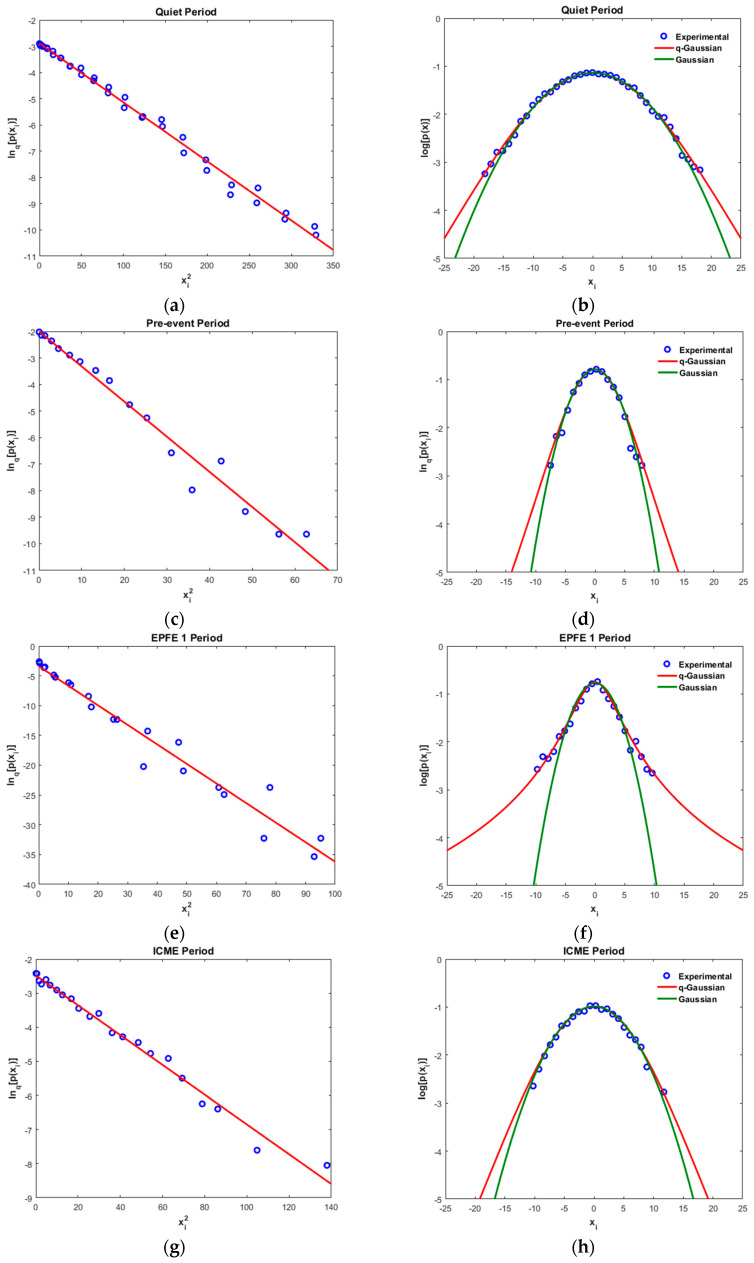

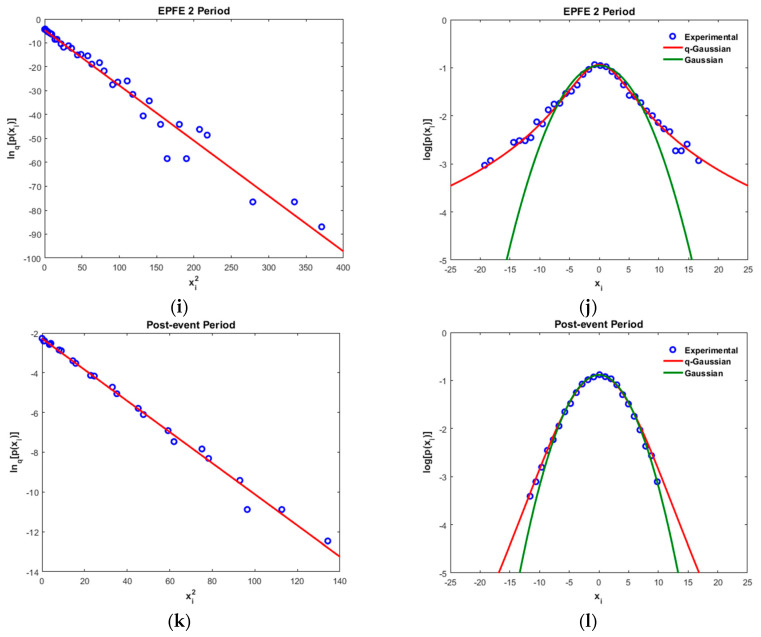
The index $q_{stat}$ for energetic particle intensities (312–555 keV). Figures in the left column shows linear correlation, while figures in the right column shows the PDF, with q-Gaussian function that fits for each period: (**a,b**) Quiet period; (**c,d**) pre-event period; (**e,f**) EPFE 1 period; (**g,h**) ICME period; (**i,j**) EPFE 2 period; (**k,l**) post-event period.

In Figure 10 we present the results concerning Tsallis statistics for the energetic ion intensity TMS and for all periods. In particular, in the left column of Figure 10 we present for all TMS, the best linear correlation (red line) between $\ln_{q}\left[p\left(x_{i}\right)\right]$ (open blue circles) and ${\left(x_{i}\right)}^{2}$ while in the right column of Figure 10 we present the difference in long tails between the q-Gaussian (red line) and the Gaussian PDF (green line), in a $\log\left[p\left(x_{i}\right)\right]$ vs $x_{i}$ graph. The open blue circles correspond to the experimental detrend TMS. In Table 5 and Table 6 presented the values that used to estimate the q-Gaussian distribution, for both energetic ion intensity and magnetic field TMS.

In all cases, the value Tsallis $q_{stat}>1$ suggests the presence of long-range interactions, a distinctive property of open non-equilibrium systems, with underlying dynamics characterized by non-Gaussian (q-Gaussian) distributions. It is the maximization of Tsallis entropy, which leads to the observed q-Gaussian distribution in contrast to BG formalism, which yields exponential equilibrium distributions. In addition, Tsallis $q_{stat}$ for the EPFE periods is much greater than the corresponding quiet, pre and post event, and ICME periods, indicating that in the EPFE periods the dynamics have a stronger sub-additive, non-extensive character, in both cases of energetic ion intensity and magnetic field TMS. Additionally, the κ index of kappa distribution is connected to the $q_{stat}$ index. Hence, through Equation (A14) the κ index for the six periods of energetic ion intensity and magnetic field TMS, was calculated to be κ=12.50 for quiet period, κ=9.09 for pre-and post-event periods, κ=14.29 for the ICME period, κ=2.13 for the EPFE 1 period, and κ=1.75 for the EPFE 2 period (Table 5). Similarly, for the magnetic field TMS the κ index was found to be κ=2.17 for quiet period, κ=1.72 for pre-event period, κ=1.39 for the EPFE 1 period, κ=1.75 for the ICME period, κ=1.23 for the EPFE 2 period, and κ=1.43 for post-event period (Table 6).

As we describe next, the kappa index describes the energy spectrum probability distribution. According to Equation (A14), as $q_{stat}$ increases the kappa index decreases while the probability distribution obtains higher values for higher energies. This means that the energy probability distribution obtains a stronger heavy tail profile as the non-extensivity and multifractality of the system is strengthened. This character of the energy probability distribution is described by Equation (19) in the discussion section. This is in agreement with our observations as the $q_{stat}$ and kappa indices correspond to higher probability values at the same energies when they are compared between those periods. For higher values of $q_{stat}$ the density of particles of energy E is higher. This can be explained through the kappa distribution of energies, which are caused by optimization of the Tsallis q-entropy [80,81,82].

(d) Evolution of q-triplet

**Figure 11:**
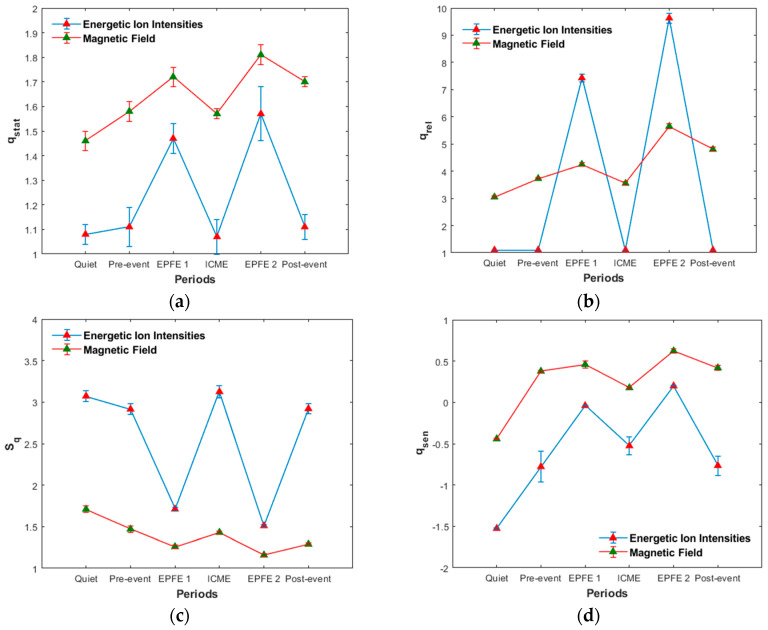
Variation of Tsallis q-triplet during the event: (**a**) $q_{stat}$ index; (**b**) $q_{rel}$ index; (**c**) q-entropy maximum $S_{q}$; (**d**) $q_{sen}$ index.

In Table 5 and Table 6, and Figure 11, we present the evolution of non-extensive statistics from period one to period six, for both energetic ion intensity and magnetic field TMS.

Concerning the energetic ion intensity measurements, the $q_{stat}$ values for periods quiet, pre-event, ICME, and post-event, remain near the value 1 but are clearly different than the Gaussian value of $q_{stat}=1$. As concerns the magnetic field measurements, the $q_{stat}$ parameter for all periods is clearly different, much higher than the value 1. From period 1 to period 6, we can see a general tendency for increase of the $q_{stat}$ index. The maximum values of $q_{stat}$ was found for EPFE periods, with higher value for EPFE 2 period (Figure 11a). The indices $q_{sen}$ and $q_{rel}$ follows the variation of $q_{stat}$ index for both kind of measurements (Figure 11b). For the energetic ion intensity measurements, the value of $q_{rel}$ index for periods quiet, pre-event, ICME, and post-event it was not possible to be calculated exactly as the relaxation process happens very quickly. For this reason, we estimate that the values of $q_{rel}$ index are higher than the Gaussian value of 1 but near at this value, taking into consideration the $q_{stat}$ index, Flatness coefficient, and p-model parameter. As concern the values of entropy $\left(S_{q}\right)$ and the rate of entropy production $\left(dS_{q}/dt\right)$, we observe similar behavior between energetic ion intensity and magnetic field measurements (Figure 11c). However, comparing the $S_{q}$ and $dS_{q}/dt$ values with $q_{stat}$ index, we observe the opposite behavior. Lower values observed during EPFE periods for both kind of measurements, and are even lower in the EPFE 2 compared to the EPFE 1 period. This profile of evolution of entropy, indicates the existence of distinct NESS of the SW plasma. All these results presented in Table 5 and Table 6, and Figure 11, clearly indicate a phase transition process between distinct NESS of the SW plasma during the SMIs events.

**Figure 12:**
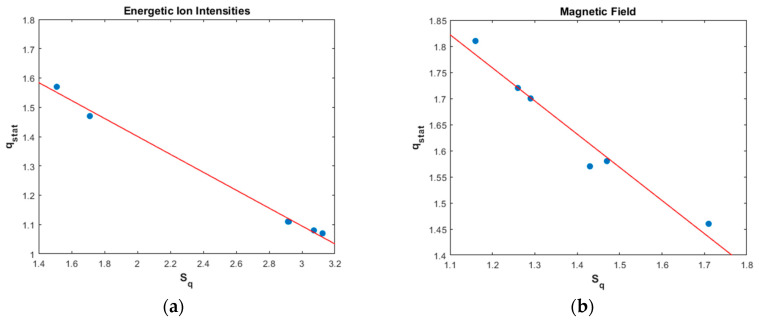
Linear correlation between q-entropy and $q_{stat}$: (**a**) For energetic ion intensity TMS; (**b**); for magnetic field TMS.

In Figure 12, we present the linear correlation between q-entropy $S_{q}$ and $q_{stat}$ for energetic ion intensities (Figure 12a) and for the magnetic field (Figure 12b). As we can see, there is a negative correlation between $S_{q}$ and $q_{stat}$, for energetic ion intensity TMS for magnetic field TMS. The value of $S_{q}$ corresponds to the NESS. As shown in Figure 12, the q-entropy maximum clearly decreases for EPFE periods, and suggests that the NESS becomes more organized, which means that fewer effective degrees of freedom are available to the dynamics as the entropy decreases. This is in accordance with the basic theoretical framework of Tsallis theory and our previous results concerning the $q_{stat}$ parameter, which was found to increase in EPFE periods: As $q_{stat}$ increases, the long-range correlations become stronger causing the decrease of the entropy.

### 5.3. Correlation Dimension Estimation

**Figure 13:**
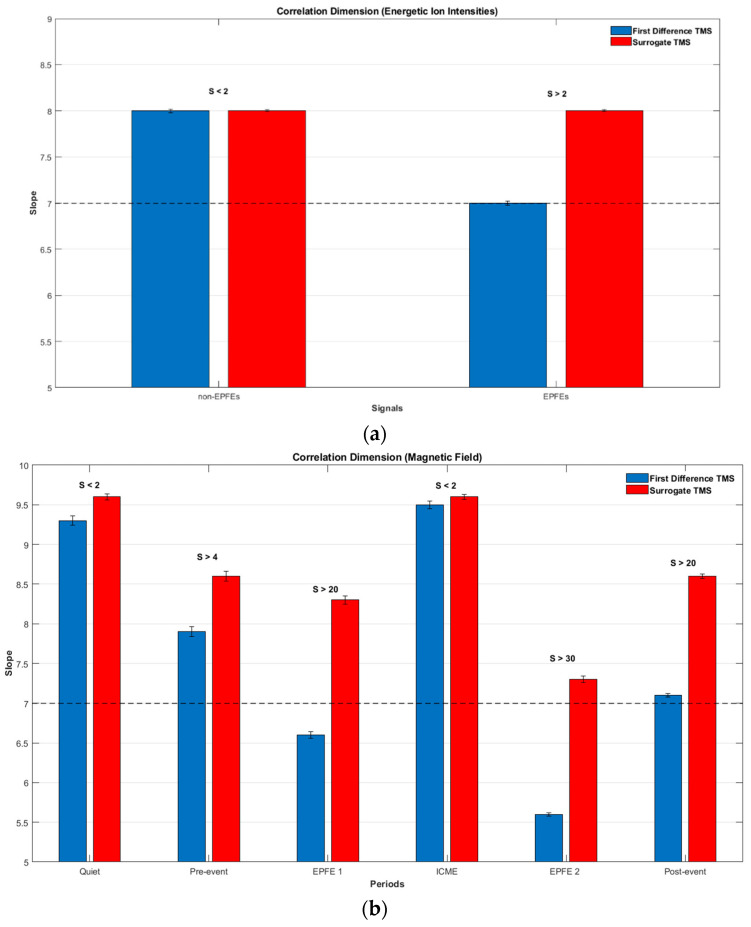
The slopes of the correlation integral as a function of the radius *r*. Blue bar corresponds to the first difference TMS and red bar corresponds to its surrogate data. (**a**) For energetic ion intensities during the EPFE periods (EPFEs) and for the rest of the periods (Non-EPFEs); (**b**) for the magnetic field TMS for the six periods.

The Correlation Dimension of the energetic particles and magnetic field TMS was estimated for their first differences (Figure 13). Concerning the energetic particle fluxes, due to the small number of data and because the algorithm of estimation of the Correlation Dimension works faithfully for signals of length $N>6\times 10^{3}$, we created two different signals. The first one (Non-EPFEs) includes the quiet, pre-event, ICME, and post-event periods, and the second one (EPFEs) includes the EPFE periods. Finally, for both signals, we estimated the Correlation Dimension. Figure 13a presents the Correlation Dimension for the energetic ion intensity TMS, estimated for the reconstructed phase space with dimension *D* = 8, as well as for the corresponding surrogate data. For the estimation of the Correlation Dimension the criteria of Theiler were used, excluding *w* temporal correlated neighbor states, where *w* was taken to be higher than the decorrelation time. In particular, the Correlation Dimension was estimated for *D* = 8, τ=3 and *w* = 10.

Our results clearly reveal the reduction of the phase space dimensionality during the EPFE periods of energetic ion intensity TMS, as well as clearly discriminated from their surrogate data as the significance *S* of the statistics was found to be higher than 2. In particular, for the energetic ion intensity TMS, due to EPFE periods, the Correlation Dimension was bound to be D≈7±0.05, while for the rest periods it was found to be D≈8±0.04 with no discrimination from surrogate data, as well as the significance *S* was found to be smaller than 2. Similar results were obtained for the magnetic field TMS. Figure 13b and Table 7 presents the Correlation Dimension for the magnetic field magnitude for each period, estimated for the reconstructed phase space with dimension *D* = 10, τ=5 and *w* = 10, as well as for the corresponding surrogate data. The results clearly reveal reduction of the phase space dimensionality during the EPFE periods of magnetic field TMS. In particular, the periods pre-event, EPFE 1, EPFE 2, and post-event, are clearly distinct from their surrogate data as the significance *S* of the statistic was found to be much higher than 2, while for the quiet and ICME period the significance *S* was found to be smaller than 2. Especially, in Figure 13b we can observe different turbulence states in the SW plasma during SMIs events. During quiet and ICME periods, where the surrogate data cannot be discriminated from the original TMS and the slope shows high Correlation Dimension the turbulent state is weak, while in the rest of the periods the significance of discrimination is higher than 2 and the slopes indicate decreasing of the Correlation Dimension. This characteristic implies the strengthening of the turbulence state. Moreover, in the EPFE periods the significance of discrimination becomes very high and the reduction of dimensionality is also higher, a fact that can be understood as an outcome of the fully developed turbulence state. The above description reveals the corresponding variation of the SW plasma self-organization character.

### 5.4. Hurst Exponent Estimation

**Figure 14:**
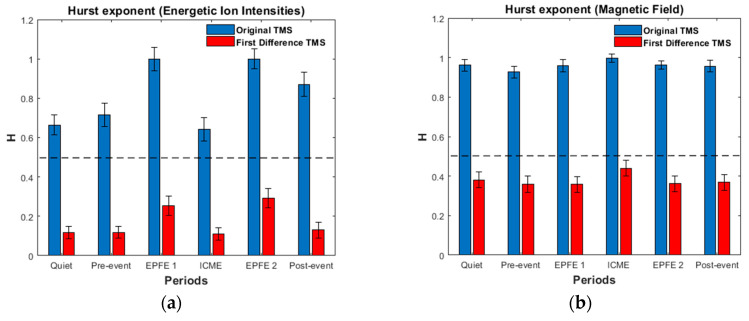
The Hurst exponent for the original signal (blue bar) and for the first difference signal (red bar): (**a**) For energetic ion intensity TMS; (**b**) for magnetic field TMS.

The Hurst exponent was estimated, for both cases of the original and the First Difference (FD) signals, also for energetic ion intensity and magnetic field TMS. Figure 14 presents the estimated values of the Hurst exponent. As can be observed, the original signals (blue bars) were found to be related for both cases of the energetic ion intensity and the magnetic field TMS with persistent (super-diffusion) random walk process, as the Hurst exponent attain values higher than 0.5, corresponding to the normal diffusion process. In contrast, the first difference signals (red bars) in both cases of the energetic ion intensity and the magnetic field TMS, were found to correspond to anti-persistent (sub-diffusion) random walk process, as the value of the Hurst exponent was found to be smaller than 0.5. Moreover, for the energetic ion intensity TMS there is noticeable differentiation at the values of Hurst exponent during the EPFE periods, where the energetic particle fluxes increase. For the original signals of the energetic ion intensities, the value further increases to values much higher than 0.9, while for the first difference TMS it increases higher than 0.25. On the other hand, for the original signals of magnetic field the values of the Hurst exponent for all periods are higher than 0.9, while for the first difference signals the values stay close to 0.4. In the following tables, we summarize the Hurst exponent for original and first difference TMS for the energetic ion intensities (Table 8) and the magnetic field (Table 9).

## 6. Summary of Data Analysis

In this study, we analyzed the statistical features of various energetic ion intensity and magnetic field measurements from the STEREO A spacecraft. The data were chosen properly to study different time periods focused on multiple ICME interactions with the rippled HCS detected on November 2007. The time periods were analysed and were sorted out as quiet, pre-event, EPFE 1, ICME, EPFE 2, and post-event periods. Significant differences in statistical features were found, indicating important variations of the energetic ion dynamics. In particular, we established:

- Concerning the Flatness coefficient:
  ○ For the energetic ion intensities Flatness, the analysis showed two distinct characters between the periods EPFE 1 and EPFE 2 and the other periods (quiet, pre-event, ICME, post-event). During the EPFE periods a strong non-Gaussian character was clearly observed, while during the rest of the periods a weak non-Gaussian character was observed.
  ○ For the magnetic field Flatness, a non-Gaussian character observed during the whole event with clear enhancement of coefficient during pre-event, EPFE 1, EPFE 2, and post-event periods. However, a stronger non-Gaussian character was observed during the pre-event, EPFE 2 and post-event periods in contrast to the EPFE 1 period.
- Concerning the Tsallis q-triplet:
  ○ The non-extensive character of the SW statistics mirrored in the q-triplet of Tsallis, was present during all the periods, since the q-triplet values were found to be different than the value $q_{sen}=q_{rel}=q_{stat}=1$ corresponding to the Boltzmann–Gibbs extensive statistics. This result, was observed for both the energetic ion intensity and magnetic field measurements. However, the non-extensive character of SW plasma was found to be stronger during the EPFE 1 and EPFE 2 periods, for both cases, as the values of q-triplet parameters $\left(q_{sen},\;q_{rel},q_{stat}\right)$ were found to be much higher than the other periods.
  ○ The multifractal plasma character mirrored at the $f\left(\alpha \right),\Delta D_{\bar{q}}$ functions was observed during all the periods for both cases of energetic ion intensities and magnetic field. However, during the two critical periods (EPFE 1 and EPFE 2) with strong energetic particle flux enhancements the multifractal character was found to be stronger than in the other periods, for both kind of measurements.
  ○ The generalized dimensions $D_{\bar{q}}$ for $\bar{q}>0$ were found to decrease as we pass from the quiet period to the next periods. Also, the generalized dimensions $D_{\bar{q}}$ for $\bar{q}>0$ was found to be lower in the EPFE 2 than the EPFE 1 period, for both energetic ion and magnetic field TMS.
  ○ The intermittent turbulence state of SW plasma was present during all the periods as the parameter of the p-model, was found to be higher than the value *p* = 0.5 corresponding to the K41 Kolmogorov’s theory of homogenous space filling turbulence. However, the intermittent character of SW turbulence was found to be stronger during periods EPFE 1 and EPFE 2 for both kind of measurements, as the value of p-model increases to higher values.
  ○ The Tsallis q-entropy value and the rate of q-entropy production were found to decrease during the EPFE periods, for both the energetic ion and magnetic field measurements. In particular, for period EPFE 2 the parameters were found to be lower than those of the period EPFE 1.
  ○ Non-Gaussianity, non-extensivity, multifractality, and intermittent turbulent character of the SW plasma becomes clearly stronger during the period EPFE 2 than the EPFE 1 period, for both energetic ion intensity and magnetic field TMS. In other words, the Flatness coefficient, the Tsallis q-triplet parameters, the $\Delta D_{\bar{q}}$ and the p-model parameters were found to obtain higher values during the period EPFE 2 than the period EPFE 1. Also, the self-organization process was found to be stronger in EPFE 2 period than the EPFE 1 period, as the reduction of the dimension was found to be higher for EPFE 2.
- Concerning the Correlation Dimension:
  ○ The Correlation Dimension corresponding to reconstructed dynamics of energetic ion intensity TMS, was found to decrease noticeably at value ≈7 and at the value ≈6.5 and ≈5.5 for the magnetic field during the periods EPFE 1 and EPFE 2, respectively. For energetic ion intensities, the observed reduction of dimensionality and the discrimination from surrogate TMS reveals a self-organization process of the underlying dynamics.
  ○ For the magnetic field, the discrimination with surrogate data was significant for all the periods, except the quiet and ICME periods, while for the energetic ion intensities the significance of discrimination was observed only during periods EPFE 1 and EPFE 2. Moreover, the estimation of Correlation Dimension of original and surrogate TMS reveals different states of SW plasma turbulence, corresponding to different order of self-organization process. In the EPFE periods we observe fully developed turbulence, while in the rest of the periods we observe early and intermediate turbulence states.
- Concerning Hurst exponent:
  ○ The anomalous diffusion character of the energetic ion intensities and magnetic field random walk process was present for all the observed periods as the Hurst exponent was found clearly different from the value *H* = 0.5, which corresponds to the normal (Gaussian) diffusion process. Moreover, the estimation of the Hurst exponent showed persistent (super-diffusion) anomalous diffusion for both the original TMS (energetic ion intensities and magnetic field) since the Hurst exponent was estimated to be much higher than the value 0.5. On the other hand, the Hurst exponent estimated for the first difference TMS for both kind of measurements, was found to be much lower than the value 0.5, revealing anti-persistent (sub-diffusion) anomalous diffusion process.
  ○ As the system deviates more and more further from Gaussian states, the anomalous diffusion, self-organization, multifractality, and non-extensivity increases simultaneously. This indicates that the Levy flight character of the random walk process is strengthened, as well as the anomalous (fractional) acceleration efficiency.

## 7. Discussion

In this study, we have analyzed observations of energetic particle flux enhancements detected in the SW by the STEREO-A spacecraft in November 2007 during the interaction of an ICME with the rippled and highly disturbed HCS. To understand the properties of energetic particles and in order to reveal a mechanism of particle acceleration, we used various diagnostic tools for the analysis of energetic particle measurements in relation with a non-extensive statistical theory of Tsallis, especially the Tsallis q-triplet parameters. As the underlying physical mechanism of SMIs structures and development of energetic particle population includes self-consistent interaction of particles and magnetic field, we analysed also magnetic field measurements during the SMI event. This analysis clearly showed the non-extensive character of the charged particle statistics and the far-from-equilibrium thermodynamic behavior of the energetic particle populations observed in association with magnetic islands in the SW. The behavior of energetic ion intensity and magnetic field TMS, was found to be almost similar for all the periods, indicating a physical connection of the underlying dynamics in both cases. Especially, during EPFE periods we observed simultaneously for both kind of measurements, the following characteristics:

- Enhancement of multifractal and intermittent turbulent character
- enhancement of the non-extensivity character
- strengthen of the self-organization process
- reduction of dimensionality
- reduction of optimum q-entropy $S_{q}$
- topological SW phase transition process

The above characteristics, are mirrored at the changes of the q-triplet, the singular spectra f(α), the generalized dimension spectrum $D_{\bar{q}}$, and the p-model parameter.

Moreover, the multifractal and intermittent turbulence character of the magnetic field measurements reveals spatial multifractal topology in the distribution of the magnetic field intensities and magnetic field energy in the physical space. Similarly, the multifractal and intermittent turbulence of the energetic ion intensity measurements indicates also the existence of multifractal topology in the spatial distribution of particle densities in the physical space. The term multifractal topology used here for the particle spatial density distributions, means the multifractal structure of particle density distributions described as a multifractal random continuum field in accordance with Milovanov [83] and Tarasov [55,84,85].

First of all, we must notice the faithful agreement of the results obtained in this study with the theoretical framework discussed in Section 2 and Appendix A. Specifically, the SW system displays a nonlinear dynamic scenario, according to which for SMIs events the SW plasma undergoes a phase transition from a weak non-Gaussian and weak non-extensive turbulent state to a strongly non-Gaussian and fully developed intermittent turbulence state. During the fully developed and strong non-extensive state, the plasma system develops self-consistently multifractal magnetic field and energetic particle structures including self-organization, anomalous diffusion, intermittent turbulence, long-range correlations, non-extensivity, reduction of dimensionality, and fractional acceleration mechanisms. All of these characteristics constitute the self-consistent holistic manifestation through the development of long-range correlations of the non-equilibrium SW plasma phase transition. Moreover, this holistic complex plasma dynamics is the manifestation of a generalized entropy principle according to which space plasma particles and fields cooperate self-consistently for the optimization of Tsallis q-entropy function.

According to the summary presented in Section 6, we have obtained novel and significant results concerning the non-extensive intermittent turbulence and multifractal character of the energetic ion flux intensity and magnetic field measurements during periods comprising all states of the SW during the passage of an ICME highly disturbing the surrounding plasma. It is clear that both the energetic ion intensity and magnetic field measurements have shown two distinct characters of the SW plasma dynamics: One kind of dynamics can be characterized as weak non-Gaussian or weak non-extensive, while the second kind (corresponding to periods with strong enhancement of energetic particle fluxes), is characterized by a strongly non-Gaussian and non-extensive dynamics. In the second kind of dynamics, the multifractal and intermittent-anomalous character, as well as the self-organization (reduction of dimensionality) process of the SW plasma system, were found to be highly developed. These results clearly indicate a SW phase transition process mirrored at both the energetic ion intensity and magnetic field dynamics. This phase transition is a topological phase transition process, as the topology of the phase space of the SW plasma dynamics changes. This change is revealed by the change of the parameters of Tsallis q-triplet, p-model, Flatness coefficient, Correlation Dimension. Also, the SW topological phase transition process includes topological changes in the fractal distribution of particle densities and magnetic field intensities in the physical space. The spatial fractal topology of the magnetic field and energetic ion distributions is mirrored at the fractional topology and multifractal structuring of the dynamical phase space.

Our study also clearly showed the physical connection between the multifractal character of the energetic ion intensity and magnetic field measurements, since both of them are observed to increase simultaneously passing from the quiet to the EPFE periods. This relation of magnetic field and energetic particle structures must be testified further with an extended statistical analysis of many SMIs events. After all, it is physically correct, in parallel with the turbulent and multifractal dynamics of the magnetic field to suppose the existence of an induced multifractal electric field distribution which can produce the acceleration of charged particles according to [1]. More specifically, the intermittent turbulence character of the magnetic field corresponds to spatiotemporal magnetic inhomogeneities and magnetic fluctuations, which can also induce spatiotemporal inhomogeneities and fluctuations of the induced electric field. This can produce the fractional acceleration of particles [54,80] and the multifractal structure of the energetic particle fluxes. The fractional acceleration of particles is connected with the anomalous diffusion and anomalous random walk-Levy flights of particles in the multifractal turbulent environment of magnetic and electric fields. The intermittent turbulence of the magnetic and the induced electric field, parallel with anomalous random walk of plasma particles, can be described by the extension of MHD theory and the underlying kinetic theory to fractional MHD theory and fractional kinetic theories [37,50,80,84,86,87,88,89]. These concepts support the idea of stochastic particle acceleration (or re-acceleration) in dynamical magnetic islands, confirming that the nature of the acceleration process is in fact the result of the induced electric field fractional characteristics, in accordance with the magnetic field distribution. In accordance to this, it is quite possible that different mechanisms leading to particle acceleration co-operate in the closed magnetic field configurations, i.e., magnetic islands can experience merging, contraction and magnetic field turbulent reconnection that accelerates particles [45,48,49,50,51]. This can be thought as a kind of resonant pumping in HCS ripples that can energize particles from keV to MeV energies as well [90].

Moreover, the non-Gaussian character of Tsallis q-triplet estimated for the energetic particle intensities and magnetic field distributions observed during EPFE periods, indicates the existence of q and kappa energy distributions of the energetic particle population and the magnetic field intensity. All these characteristics can be explained by the extremization of Tsallis q-entropy. That is the maximization of Tsallis q-entropy, leads to the development of complex SMIs structures including the multifractal-intermittent turbulence structures of magnetic field and energetic particles. Moreover, this complex plasma structures must include also self-consistently the bulk plasma flow intermittent turbulence and the hypothesized here induced electric field that accelerate particles.

The non-equilibrium plasma states of fields and particles thermodynamically correspond to the extremization of Tsallis q-entropy under appropriate constraint conditions [32]. Tsallis q-exponential probability distributions of the energy take a form of kappa distributions of two main types as follows:

(19) $$P^{\left(1\right)}\left(\varepsilon_{\kappa }\right)\sim {\left[1+\frac{1}{\kappa^{*}}\cdot \frac{\varepsilon_{\kappa }-U\left(T^{*},\kappa^{*}\right)}{k_{B}T^{*}}\right]}^{-\kappa^{*}},\;\;\;\;P^{\left(2\right)}\left(\varepsilon_{\kappa }\right)\sim {\left[1+\frac{1}{\kappa }\cdot \frac{\varepsilon_{\kappa }-U\left(T\right)}{k_{B}T}\right]}^{-\kappa -1},$$

which especially occurs in the case of energetic (nonthermal) particle populations. In Equation (19), U is the mean kinetic energy $U=\langle \varepsilon_{\kappa }\rangle$.

According to Livadiotis and McComas [81,82], the Tsallis q-distributions of the energetic particles and their kappa distributions are linked through Equation (19). Our results clearly reveal a non-Gaussian energy spectrum including heavy tails for the probability distributions of the energetic particle population. The anomalous-fractal topology of the magnetic field corresponding to strong self-organization of the SW plasma, causes a correspondent induced electric field topology including local accelerating regions. This theoretical concept is in agreement with fractal topology of magnetic energy dissipation. Hence, the existence of local accelerating sources is the manifestation of the global self-consistent dynamics of magnetic fields and particles. 

The indicated topological phase transition of the SW plasma system can be related with NESS [37] corresponding to local minimums of plasma Free energy and local maximum of plasma entropy. This is in agreement with the results of this study, as the Tsallis q-entropy $S_{q}$ values and the rate of q-entropy $dS_{q}/dt$ were found to decrease during the EPFE periods where the phase transition process takes place. The non-equilibrium (quasi)stationary plasma states are caused as the plasma system tries to extremize its q-entropy in accordance with the general entropy principle of statistics [32]. The extremization of plasma Tsallis q-entropy function produces the q-Gaussian probability distribution functions of magnetic field and energetic ion intensity measurements observed in this study. Moreover, the phase transition of plasma system from Gaussian to near-Gaussian and further to strongly non-Gaussian physical NESS, is related to the extension of the classical CLT to the q-CLT which explains the non-Gaussian character of the q-triplet parameters, as we have observed here. The phase transition process of the SW plasmas during SMIs events is also in agreement with the non-equilibrium renormalization group theory (RGT) of Chang [91].

The multifractal structuring of the SW magnetic field and its intermittent turbulence character indicates inhomogeneous local energy plasma dissipation in accordance with the Cantor multiplicative process and as indicated by the p-modeling of the magnetic field multifractal character. The correspondent multifractal and intermittent character of energetic particle fluxes can be explained by the magnetic field intermittent turbulence, which can be related with local sources of induced electric field. That is the intermittent character of the magnetic field distribution must be related with the intermittent character of energetic particle fluxes through the inhomogenous spatiotemporal distributions of the hypothesized induced electric field sources. Moreover, as the non-extensivity and self-organization-reduction of dimensionality, of the SW magnetic field dynamics enhanced passing from EPFE 1 to EPFE 2 period, we can explain the corresponding higher fluxes of energetic particles at period EPFE 2 than the EPFE 1 period. From this point of view, the singularity spectra f(α) and the spatial distribution of singularities α of magnetic field and energetic particles, are self-consistently related with the indicated singularity spectra f(α) and the spatial distribution of singularities α of the hypothesized here induced electric field through the maximization of Tsallis q-entropy.

The results of this study based on the non-extensive statistical theory of Tsallis, indicate the necessity of future work including the study of non-equilibrium energy spectra of SW energetic particles, as well as the study of non-extensive character of bulk MHD plasma parameters in relation with SMIs phenomena.

## Figures and Tables

**Table 1:** Flatness coefficient for energetic ion intensity TMS.

Parameter	Period 1 (Quiet)	Period 2 (Pre-event)	Period 3 (EPFE 1)	Period 4 (ICME)	Period 5 (EPFE 2)	Period 6 (Post-Event)
F (Original TMS)	3.10 ± 0.07	2.74 ± 0.07	4.81 ± 0.24	3.05 ± 0.17	5.04 ± 0.84	3.17 ± 0.15
F (FD TMS)	3.07 ± 0.06	2.85 ± 0.06	3.91 ± 0.16	2.95 ± 0.19	4.66 ± 0.73	3.05 ± 0.11

**Table 2:** Flatness coefficient for magnetic field TMS.

Parameter	Period 1 (Quiet)	Period 2 (Pre-event)	Period 3 (EPFE 1)	Period 4 (ICME)	Period 5 (EPFE 2)	Period 6 (Post-Event)
F (Original TMS)	10.45 ± 1.15	15.27 ± 1.96	10.58 ± 1.55	8.17 ± 0.85	14.72 ± 1.81	16.09 ± 2.09
F (FD TMS)	7.26 ± 0.37	16.31 ± 2.56	10.02 ± 1.50	6.98 ± 0.79	14.22 ± 1.74	14.78 ± 1.98

**Table 3:** Parameters estimated from singularity spectrum for energetic ion intensity TMS.

Parameter	Period 1 (Quiet)	Period 2 (Pre-Event)	Period 3 (EPFE 1)	Period 4 (ICME)	Period 5 (EPFE 2)	Period 6 (Post-Event)
α_0_	1.010	1.023	1.087	1.028	1.103	1.022
*A*	1.046 ± 0.008	1.006 ± 0.084	0.978 ± 0.035	0.857 ± 0.068	1.148 ± 0.007	0.849 ± 0.042
Δα	0.386 ± 0.001	0.546 ± 0.002	0.936 ± 0.001	0.656 ± 0.002	1.066 ± 0.001	0.573 ± 0.002
Δ*Dq*	0.180	0.313	0.641	0.425	0.822	0.327
*q_sen_*	−1.527 ± 0.007	−0.779 ± 0.187	−0.040 ± 0.005	−0.526 ± 0.110	0.199 ± 0.004	−0.766 ± 0.117
p-model	0.560	0.587	0.649	0.606	0.671	0.589

**Table 4:** Parameters estimated from singularity spectrum for magnetic field TMS.

Parameter	Period 1 (Quiet)	Period 2 (Pre-Event)	Period 3 (EPFE 1)	Period 4 (ICME)	Period 5 (EPFE 2)	Period 6 (Post-Event)
α_0_	1.038	1.128	1.238	1.038	1.219	1.148
*A*	0.935 ± 0.006	1.836 ± 0.071	1.689 ± 0.233	1.609 ± 0.028	1.592 ± 0.207	1.257 ± 0.124
Δα	0.683 ± 0.001	1.062 ± 0.001	1.315 ± 0.010	0.851 ± 0.001	1.478 ± 0.010	1.281 ± 0.010
Δ*Dq*	0.430	0.821	1.003	0.559	1.142	0.955
*q_sen_*	−0.442 ± 0.010	0.377 ± 0.010	0.459 ± 0.045	0.177 ± 0.012	0.623 ± 0.030	0.418 ± 0.031
p-model	0.609	0.669	0.705	0.621	0.720	0.693

**Table 5:** Summary of non-extensive statistics for energetic ion intensity TMS.

Parameter	Period 1 (Quiet)	Period 2 (Pre-Event)	Period 3 (EPFE 1)	Period 4 (ICME)	Period 5 (EPFE 2)	Period 6 (Post-Event)
q*_stationary_*	1.08 ± 0.04	1.11 ± 0.08	1.47 ± 0.06	1.07 ± 0.07	1.57 ± 0.11	1.11 ± 0.05
q*_sensitivity_*	−1.527 ± 0.007	−0.779 ± 0.187	−0.040 ± 0.0.005	−0.526 ± 0.110	0.199 ± 0.0.004	−0.766 ± 0.117
q*_relaxation_*	-	-	7.43 ± 0.14	-	8.63 ± 0.18	-
S*_q_*	3.06 ± 0.07	2.93 ± 0.06	1.72 ± 0.02	3.13 ± 0.07	1.51 ± 0.01	2.92 ± 0.06
dS_q_/dt	4786.00 ± 581.00	386.80 ± 40.50	40.20 ± 3.05	174.30 ± 15.30	20.97 ± 1.43	372.50 ± 35.08
κ	12.50	9.09	2.13	14.29	1.75	9.09

**Table 6:** Summary of non-extensive statistics for magnetic field TMS.

Parameter	Period 1 (Quiet)	Period 2 (Pre-Event)	Period 3 (EPFE 1)	Period 4 (ICME)	Period 5 (EPFE 2)	Period 6 (Post-Event)
q*_stationary_*	1.46 ± 0.04	1.58 ± 0.04	1.72 ± 0.04	1.57 ± 0.02	1.81 ± 0.04	1.70 ± 0.02
q*_sensitivity_*	−0.442 ± 0.010	0.377 ± 0.010	0.459 ± 0.045	0.177 ± 0.012	0.623 ± 0.030	0.418 ± 0.031
q*_relaxation_*	3.05 ± 0.04	3.73 ± 0.05	4.25 ± 0.06	3.55. ± 0.06	5.65 ± 0.09	4.80 ± 0.08
S*_q_*	1.71 ± 0.04	1.47 ± 0.04	1.26 ± 0.02	1.43 ± 0.02	1.16 ± 0.02	1.29 ± 0.02
dS_q_/dt	121.80 ± 18.29	12.50 ± 1.31	10.34 ± 0.91	20.85 ± 2.02	7.01 ± 0.62	11.23 ± 1.15
κ	2.17	1.72	1.39	1.75	1.23	1.43

**Table 7:** Correlation Dimension for magnetic field TMS.

Parameter	Period 1 (Quiet)	Period 2 (Pre-Event)	Period 3 (EPFE 1)	Period 4 (ICME)	Period 5 (EPFE 2)	Period 6 (Post-Event)
D (FD TMS)	≈ 9.3 ± 0.06	≈ 7.9 ± 0.06	≈ 6.6 ± 0.04	≈ 9.5 ± 0.05	≈ 5.6 ± 0.02	≈ 7.1 ± 0.02
D (Surrogate Data)	≈ 9.6 ± 0.04	≈ 8.6 ± 0.06	≈ 8.3 ± 0.05	≈ 9.6 ± 0.03	≈ 7.3 ± 0.04	≈ 8.6 ± 0.03

**Table 8:** Hurst exponent for energetic ion intensity TMS.

Parameter	Period 1 (Quiet)	Period 2 (Pre-Event)	Period 3 (EPFE 1)	Period 4 (ICME)	Period 5 (EPFE 2)	Period 6 (Post-Event)
H (Original TMS)	0.66 ± 0.05	0.72 ± 0.06	1.00 ± 0.06	0.64 ± 0.06	1.00 ± 0.05	0.87 ± 0.06
H (FD TMS)	0.12 ± 0.03	0.12 ± 0.03	0.25 ± 0.05	0.11 ± 0.03	0.29 ± 0.05	0.13 ± 0.04

**Table 9:** Hurst exponent for magnetic field TMS.

Parameter	Period 1 (Quiet)	Period 2 (Pre-Event)	Period 3 (EPFE 1)	Period 4 (ICME)	Period 5 (EPFE 2)	Period 6 (Post-Event)
H (Original TMS)	0.96 ± 0.03	0.96 ± 0.03	0.96 ± 0.03	0.99 ± 0.02	0.96 ± 0.02	0.96 ± 0.03
H (FD TMS)	0.38 ± 0.04	0.36 ± 0.04	0.36 ± 0.04	0.44 ± 0.04	0.36 ± 0.04	0.37 ± 0.04

## Appendix A

The development of SW SMIs structures that can be observed in situ by spacecraft instrumentations, constitute an interesting and significant manifestation of non-equilibrium SW plasma self-organization and topological phase transition process. From the physical point of view, the motive power and cause of such a process is the general entropy principle according to which nature works to maximize the entropy value of the dynamical system. This entropic process acts self-consistently with a nonlinear interaction of particles and fields in accordance with the macroscopic nonlinear MHD theory and the microscopic kinetic plasma dynamics [11,91,92,93]. The observed SMIs non-equilibrium SW structures, are developed as NESS according to the Zelenyi and Milovanov theory [37]. In this way, space nonlinear interactions among plasma particles and fields realize these states as stationary plasma states where the Tsallis q-entropy function is maximized during the plasma self-organization process. In the following, we shortly introduce the reader of this study in the cosmos of complexity theory, where novel concepts and processes such as: non-extensivity, q-entropy principle, self-organization, topological phase transition, intermittent turbulence, non-Gaussian statistics, fractal-multifractal (multiscale) structures, strange dynamics and fractional acceleration, long-range spatiotemporal correlations, anomalous diffusion-random walk, Levy distribution and Levy flights, q-extension of central limit theorem (q-CLT), Tsallis q-triplet, etc. can be physically explained and understood [36,52,54,94].

### Appendix A.1. Space Plasmas Complexity Theory Highlights

Generally, space plasmas are strongly nonlinear distributed physical systems described macroscopically by fluid equations fulfilled with electrodynamic equations according to the well known MHD theory [11]. At or near thermodynamical equilibrium, the MHD plasma system is described statisticaly by Boltzmann-Gibbs entropy theory as well as by Boltzmann-Gauss probability distribution functions. That is the kinetic theory underlying the MHD theory includes typical normal phenomena (normal diffusion, normal random walk processes) described by normal Langevin and Fokker-Planck equations. In a deeper kinetic statistical description of the MHD plasma system, the Bogoliubov system of statistical plasma equations can be described by BBKGY (Bogoliubov–Born–Green–Kirkwood–Yvon) equations [95,96]. However, the results of this study concern the far from thermodynamic equilibrium MHD theory and the underlying kinetic and statistical theory, which now must be extended to fractional MHD and Kinetic theory [80,81,84,85,86], as well as to the non-extensive statistical theory including the q-entropy principle [32].

#### Appendix A.1.1. Deterministic Nonlinear Dynamics

The plasma MHD nonlinear system of equations belongs to the general archetypical type:

(A1) $$\frac{d\vec{X}(\vec{r},t)}{dt}=F_{\lambda }(\vec{X}),$$

where $\vec{X}$ belongs to a Hilbert space and describes the distributed electric-magnetic fields and plasma particles. The control parameter λ describing the interaction of the plasma system and its environment. The phase space of the MHD partial equations system is an infinite dimensional linear Euclidean geometrical space [53]. The type of solutions of a nonlinear infinite dimensional physical system depends upon the physical control parameter λ. As λ changes, the nonlinear dynamics develops different kinds of stationary solutions such as: stable, periodic or quasi-periodic, chaotic (strange attractor), turbulent (early of fully developed). As λ passes critical values the system reveals phase transition processes as the new solutions of the nonlinear equations correspond to different physical states and topologies in phase space or in the physical space of the distributed physical magnitudes [53,97]. The series of physical phase transitions to new solutions as λ increased, can be related by reduction of phase space dimensionality. The reduction of dimensionality means physical development of long-range correlations (spatial and temporal) which means self-organization physical processes in the system. Parallely the self-organized system phase space, can change from Euclidean to a non-Euclidean, fractal or multifractal sets of physical states [98]. Fractal sets of self-organized physical states includes discontinuities and singularities (α) described by power laws and scale symmetry relations, which correspond to physical singularities of the distributed physical magnitudes [11]. The physical discontinuities-singularities can be contained in a mixture of fractal sets of different dimensionality as it can be described by generalized dimension spectrum $D_{q}$ or the singularity spectrum f(α) which can be estimated experimentally [58,64].

#### Appendix A.1.2. Stochastic and Statistical Description of Nonlinear Dynamics

At low values regime of the control parameter λ where the attractor is a limit point (fixed point), the thermodynamical process of the nonlinear distributed dynamical system corresponds to the state of thermodynamic equilibrium. At the state of thermodynamic equilibrium or near thermodynamic equilibrium, there exists separation of the microscopic and macroscopic time scales. This mean separation of the macroscopic deterministic dynamics from the microscopically induced random fluctuations, which follow the Gaussian statistics. The stochastic character of this state corresponds to the Boltzmann-Gibbs statistical theory including the maximization of the well-known Boltzmann-Gibbs entropy function:

(A2) $$S_{BG}=-k_{B}\int f(x)\ln\left(f(x)\right)dx,$$

In this case, the system approach the thermodynamic equilibrium by normal diffusion and normal random walk processes. At higher regimes of control parameter values the nonlinear character of dynamics makes lack of time scale separation. In this case, the system reveals global scaling of dynamics, and statistics as multiscale Levy processes—Levy flights can be present causing long-range correlations in the system and non-differentiability of Levy distributions parallel with fractal-multifractal structures in the phase space or in the physical space. Levy distributions, are connected with fractional statistical or deterministic equations of the non-equilibrium dynamics where temporal and spatial derivatives obtain fractional character as the system becomes temporally and spatially correlated. On the other hand, the CLT of classical statistics obtains new generalized form [54,99,100].

The fractal structuring of phase space at states far from thermodynamic equilibrium is related with non-Gaussian anomalous diffusion processes with persistent or anti-persistent character described by the Hurst exponent (*H*), which is related with the fractional character of temporal $\left(\partial^{a}/\partial t^{a}\right)$ and spatial $\left(\partial^{\beta }/\partial x^{\beta }\right)$ derivatives through the relation H=a/2β. Gaussian (non-strange) statistics and kinetics corresponds to the value H=1/2, as a=β=1 [37]. The fractional indices (a, β) are related also to the fractal character of phase space through the relation a/β=2/(2+θ), where θ is the connectivity index of the fractal phase space set [37]. The anomalous diffusion-random walk in the fractal phase space of the dynamics corresponds to fractional Langevin and fractional Fokker-Planck equations while the mean square displacement of random variables is given by the equation:

(A3) $$<x^{2}(t)>\;\sim \;Dt^{\mu },$$

where the index μ is related to the Hurst exponent, connectivity index and fractional indices by the relation μ=2H=2/(2+θ)=α/β. For values 0≤μ<1 and 0≤H<1/2 the random walk of the anomalous diffusion process in the phase space is anti-persistent (sub-diffusive), while for values 1<μ≤2 and 1/2<H≤1 it is persistent (super-diffusive). As concerns the connectivity index θ corresponding the Hausdorff dimension of geodesic lines in the fractal phase space it takes the values θ=0 for normal diffusion (Euclidean geometry pf phase space), θ>0 for sub-diffusive process and θ<0 for super-diffusive process. The last two cases correspond to fractal sets with path-connected topology (θ>0) and path-disconnected topology (θ<0) [37].

In this framework of fractal-multifractal structure of phase space or physical space distributions, Tsallis introduced the novel generalization of extensive statistical theory of Boltzmann-Gibbs to the non-extensive statistical theory, known as non-extensive Tsallis statistical theory [32]. Non-extensive statistical theory is presented in the following and it is based to the generalization of Boltzmann-Gibbs entropy function (*S_BG_*) to the Tsallis q-entropy function (*S_q_*), depending on the parameter *q*. For thermodynamic equilibrium *q* = 1 (Gaussian statistics), while far from thermodynamic equilibrium q≠1 (non-Gaussian statistics). As the nonlinear dynamics lives far from thermodynamic equilibrium it obtains non-equilibrium stationary states where the probability distributions correspond to the maximization of Tsallis q-entropy function, in correspondence with the maximization of Boltzmann-Gibbs entropy function at the thermodynamic equilibrium, and they are described by q-exponential distribution functions [32].

After the above novel dynamical and statistical concepts, we must remark the followings. Entropy as a physical magnitude is fundamental either the system lives near thermodynamic equilibrium either it lives far from thermodynamic equilibrium. Local NESS are characterized by local maximums of the system Free energy, as Tsallis has shown [32]. However, the mathematical manifestation of the entropy physical magnitude is q-dependent in accordance with the q-mathematics described in the following. Also, the entropy principle is a fundamental physical principle as nature developing near equilibrium or far from equilibrium stationary (stable) states trying to maximize the entropy function. Near thermodynamic equilibrium the entropy maximization produces Gaussian statistical processes (Boltzmann-Gibbs entropy maximization), while far from thermodynamic equilibrium the entropy maximization produces non-Gaussian statistical processes (Levy distributions, Tsallis q-exponential distributions, etc).

Summarizing the above description, we can understand the highlights of complexity theory as follows:

- *Near thermodynamic equilibrium:* Euclidean geometry-topology of phase space, normal diffusion processes, Gaussian distributions, Boltzmann-Gibbs entropy function, and CLT.
- *Far from thermodynamic equilibrium:* Non-Euclidean fractal geometry-topology of phase space, long-range spatial-temporal correlations, topological phase transition, anomalous diffusion processes, non-Gaussian Levy processes, q-extension of CLT, self-organization and reduction of phase space dimensionality, non-extensive statistics fractional kinetic theory (fractional Langevin and Fokker-Planck equations), fractional dynamics (fractional electrodynamics, fractional fluids and MHD processes) [37,87,88,99,100].

#### Appendix A.1.3. Reconstruction of SW Dynamics by Observed Time Series

In this study, we analyse measurements of SW energetic ion intensity and SW magnetic field, obtained by STEREO A spacecraft instrumentations as the spacecraft moves in the SW bulk plasma flow reference system. These measurements characterize spatiotemporal changes of the SW magnitudes and constitute TMS, which can be used for the reconstruction of the SW dynamical phase space by using the Takens embedding theory [62]. In fact, the SW plasma dynamical state space, is described macroscopically by the SW state vector:

(A4) $${\vec{X}}_{SW}(\vec{r},t)=\left\{{\vec{X}}_{sep}(\vec{r},t),{\vec{X}}_{mf}(\vec{r},t),{\vec{X}}_{ef}(\vec{r},t),{\vec{X}}_{bp}(\vec{r},t)\right\},$$

where *SW*, *sep*, *mf*, *ef*, *bp* means correspondingly SW, solar energetic particle, magnetic field state, electric field state, bulk plasma state including bulk plasma density, velocity and temperature.

According to Takens embedding theory [62], the TMS measurements of the SW magnitude includes information for all the other magnitudes as all of them are connected through deterministic MHD plasma processes and kinetic statistical correlations. From this point of view, dynamical or statistical characteristics of the SW TMS contains correspondent characteristics of the entire system. Τhe TMS signal can be considered as the one-dimensional section of the whole phase space which is constructed by the all independent physical magnitudes of the SW system, in accordance with the global state vector ${\vec{X}}_{SW}$ of Equation (A4). The observed TMS of energetic ion intensity and magnetic field, includes information for the fractal-multifractal structure of the corresponding spatially distributed physical quantities as concerns the spatial distribution of energetic particle spatial densities and magnetic field intensity.

Also, the observed TMS of energetic ion intensity and magnetic field, includes information for the fractal-multifractal structure of the entire SW phase space in accordance to Takens embedding theory [62]. That is the singularity spectrum f(α) and generalized dimension $D_{\bar{q}}$ estimated by using the energetic particle and magnetic field signals, are the one dimensional projection of the corresponding spectra ($f\left(\alpha \right),D_{\bar{q}}$) of the SW multi-dimensional phase space corresponding spectra. In this way, we can understand the physical mirroring of the multi-dimensional SW phase space fractal-multifractal structure in its one dimensional section obtained as the spacecraft moves in the SW plasma system.

The fractal-multifractal structure of the observed signals can be described by the theory of Hölder exponents [101]. According to Hölder exponent theory, the exponent of the random field f(x) measures $h\left(x_{*}\right)$ how irregular is the field f(x) at the point $\left(x_{*}\right)$. If $h\left(x_{*}\right)\in \left[n,n+1\right]$, then f(x) is *n* times but not n+1 times differentiable at the point $\left(x_{*}\right)$. The smaller the exponent $h\left(x_{*}\right)$ is, then the random field f(x) is more irregular at this point. The singularity spectrum associated with singularities *h* (Hölder exponents) of the random field variable corresponds to a mixture of fractal sets of points (*x*), and the Hausdorff dimension *D*(*h*) of the set where the Hölder exponent is equal to *h*:

(A5) $$D(h)=d_{H}\left\{x,\;\;\;h(x)=h\right\},$$

The spectrum f(h) of the singularities {*h*} describes the multifractal structure of the random field variables. In the following, the SW multifractal spectra is described by the symbols (α,f(α)), where α are the Hölder exponents of the observed signals.

The singularity spectrum f(α) of the SW plasma is related to the spatial intermittent turbulence state of the bulk plasma flow state. The development of intermittent turbulence of the bulk plasma flow creates also spatial intermittent turbulence of the SW magnetic field mirrored at the magnetic field singularity spectrum f(α), related with the spatial distribution of local magnetic field reconnection process. That is the self-consistent development of bulk plasma flow and magnetic field turbulence state is the physical environment where magnetic reconnection-magnetic energy dissipation and acceleration of particles can happen. In this way, as concerns the singularity spectrum of the magnetic field random variable, its multifractal structure corresponds self-consistently at the multifractal magnetic field energy dissipation underlying the charged particles acceleration. Also, the multifractal structure of the magnetic energy dissipation field is mirrored at the multifractal structure of energetic particle densities distribution. That is the multifractal structure of energetic ion intensity TMS mirrors the multifractal structure of the magnetic field TMS.

Through this theoretical concepts framework, we can understand the mechanism of fractional acceleration of plasma particles, which realize anomalous random walk-Levy flights movement movements in the environment of the spatiotemporal multifractal structuring of the magnetic field. According to these theoretical concepts it is physically correct to infer the existence also of multifractal spatiotemporal structuring of an induced electric field according to the Faraday law. That is, the hypothesized induced electric field corresponds to the spatiotemporal random magnetic field distribution. That is the spatiotemporal multifractal structuring of the SW system, including self-consistently dependent multifractal structuring of fields and particle random variables, can be developed as nature realize a non-equilibrium maximization of the Tsallis q-entropy. The term non-equilibrium maximization of entropy describes non-equilibrium stationary plasma states where the plasma system entropy obtains local maxima and the plasma system and its Free energy reveals local minimums. Accepting the basic concept of non-extensive statistical theory (described in the following paragraph) the local maxima of entropy corresponds to the maximization of the Tsallis q-entropy function. This maximization of the Tsallis q-entropy function, explains physically the development of multifractal magnetic field and energetic particles anomalous random walk and anomalous diffusion-Levy flight structures. The anomalous random walk-anomalous diffusion-Levy flight changes of magnetic field and energetic particles at non-equilibrium stationary plasma states describe either the motion in the plasma phase space or the mirrored plasma changes in the physical space. This concept is in accordance with the study of Alemany and Zanette [56], where the maximization of Tsallis q-entropy function explains Levy flights jump probabilities-Levy distributions and can describe the creation of fractal structures in physical space or the phase space. Similarly, in the study of Zanette and Alemany [57], the maximization of Tsallis q-entropy function applied for random energy values can produce the anomalous diffusion-random walk profile and the anomalous diffusion exponent μ for sub-diffusive or super-diffusive processes.

### Appendix A.2. Non-Extensive Entropy (*S_q_*) and q-Triplet of Tsallis Statistics

The statistical theory of Boltzmann and Gibbs (BG) is based on molecular chaos hypothesis, related to the ergodic motion of the system in the microscopical phase space. That is the system can dynamically visit with equal probability all the allowed microscopic states. The dynamical attractor of the (BG) system dynamics can be a finite or an infinite dimensional chaotic object related with thermodynamical equilibrium. The probability distributions are Gaussians and the observed magnitudes (TMS) reveal fluctuations in accordance with normal diffusion processes [32]. The equilibrium Gaussian random dynamics corresponds to physical states of uncorrelated or locally correlated noise. TMS signals related with this kind of dynamics, even if they are subjected to nonlinear distortion [64,65] revealing non-Gaussian and correlated profile, they can identified as Gaussian processes [66,67,102]. On the other side the far from equilibrium nonlinear dynamics can reveal strong self-organization and development of bifurcation processes causing long-range correlations and strange attractors. Now the Gaussian statistics is unable to describe the fluctuations as these phenomena obey to non-Gaussian statistics, with break-down of the conditions of validity of the standard central limit theorem and the theorem of large numbers [31,32,103,104].

Any extension of a physical theory is usually related to some special type of mathematics. Non-extensive Tsallis statistical theory is connected to the q-extension of exponential and logarithmic functions and the q-extension of a Fourier transform (FT) [32]. The q-extension of mathematics underlying the q-extension of statistics is included in the solution of the nonlinear equation:

(A6) $$\frac{dy}{dx}=y^{q},\;\;\left(y(0)=1,\;q\in R\right),$$

Its solution is the q-exponential function $e_{q}^{x}$:

(A7) $$e_{q}^{x}={\left[1+(1-q)x\right]}^{1/(1-q)},$$

Analytical description of the q-exponential function for positive and negative q-values, are included in Section 3.1 of [32] and figures there in.

The q-extension of logarithmic function is the inverse of $e_{q}^{x}$:

(A8) $$\ln_{q}x\equiv \frac{x^{1-q}-1}{1-q},$$

The q-logarithm satisfies the property:

(A9) $$\ln_{q}(x_{A}x_{B})=\ln_{q}x_{A}+\ln_{q}x_{B}+(1-q)(\ln_{q}x_{A})(\ln_{q}x_{B}),$$

Tsallis, inspired by multi-fractal analysis [32] proposed that the BG entropy:

(A10) $$S_{BG}=-k\sum p_{i}\ln p_{i}=k\langle \ln(1/p_{i})\rangle ,$$

cannot describe the entire of the complexity of nonlinear dynamic systems. BG statistical theory presupposes ergodicity of the underlying dynamics in the system phase space. The complexity of dynamics is far beyond simple ergodic complexity and can be described by non-extensive Tsallis statistics based on the extended concept of q-entropy:

(A11) $$S_{q}=k\frac{1-\sum_{i-1}^{N}p{\left(i\right)}^{q}}{q-1}=k\frac{1-\int_{-\infty }^{+\infty }\left(p{\left(x\right)}^{q}dx\right)}{q-1},$$

where p(i),p(x) is the probability of occupation of state *i* for discrete state space or *x* for continuous state space and *q* is a real number. The basic entropy law of thermodynamics and Boltzmann-Gibbs kinetic and statistical theory corresponds to the monotonous increasing of entropy as the system approach the thermodynamical equilibrium state. This basic law is mirrored at the maximization (extremization) of Boltzmann-Gibbs entropy function described by Equation (A10). This leads to the well-known Gaussian probability distribution functions. Similarly, the non-extensive statistical theory of Tsallis, which include the maximization (extremization) of Tsallis q-entropy function (A11), leads to q-exponential distribution functions described by Equations (A6) and (A7). The optimizing Tsallis q-distribution function depends upon the constraints of the entropy function optimization depending if the q-mean value $\langle x_{q}\rangle$ of the q-mean value of the squared variable $\langle x^{2}\rangle {}_{q}$ is known [32].

For a system of particles and fields with short-range correlations in their immediate neighborhood, the Tsallis q-entropy $S_{q}$ asymptotically leads to BG entropy $\left(S_{BG}\right)$ corresponding to q=1. For probabilistically dependent or correlated system A and B, it can be proven that

(A12) $$\begin{array}{l} S_{q}(A+B)=S_{q}(A)+S_{q}(B/A)+(1-q)S_{q}(A)S_{q}(B/A)\\ =S_{q}(B)+S_{q}(A/B)+(1-S_{q}(B)+S_{q}(A/B) \end{array}$$

where $S_{q}\left(A\right)\equiv S_{q}\left(\left\{p_{i}^{A}\right\}\right)$, $S_{q}\left(B\right)\equiv S_{q}\left(\left\{p_{i}^{B}\right\}\right)$, $S_{q}\left(B/A\right)$ is the conditional entropy of system *B* in relation to system *A* and $S_{q}\left(A/B\right)$ is the conditional entropies of system *A* in relation to system *B*. The conditional entropies depend upon the probability values $\left\{p_{ij}\right\}$ associated with the total system A+B [32]. When the systems are probabilistically independent, then Equation (A12) changes to

(A13) $$S_{q}(A+B)=S_{q}(A)+S_{q}(B)+(1-q)S_{q}(A)S_{q}(B),$$

The first part of $S_{q}\left(A+B\right)$ is additive $S_{q}\left(A\right)+S_{q}\left(B\right)$, while the second part is multiplicative including long-range correlations supporting the macroscopic ordering phenomena. That is the multiplicative term $\left(1-q\right)S_{q}\left(A\right)S_{q}\left(B\right)$ is related with the development of long-range correlations in the system for q≠1. For q=1 the optimization of entropy leads to Gaussian probability functions which cannot describe long-range correlations. However, for q≠1 the optimization of entropy leads to Levy type and q-Gaussian PDFs, which include the possibility for development of long-range correlations, self-organization mechanisms and anomalous diffusion process. Milovanov and Zelenyi showed that the Tsallis definition of entropy is consistent with the so-called “kappa” distribution for space plasmas and other physical realizations [80,81,82,105,106,107]. They also found that the Tsallis entropy formalism can be applied to physical systems with relatively small statistical weights (Ω), where Ω is the total number of the possible (microscopic) states, while for large statistical weights the standard BG statistical mechanism is better [80]. This result means that when the dynamics of a system is attracted in a confined subset of the phase space, then long-range correlations can develop. According to Tsallis, if the correlations are either strictly or asymptotically non-existent, the BG entropy is extensive, whereas $S_{q}$ for q≠1 is non-extensive. Here the term extensive means the additivity property of entropy, while the term non-extensive means the non-additivity property of entropy through the multiplicative term $\left(1-q\right)S_{q}\left(A\right)S_{q}\left(B\right)$. Oppositely, for strongly correlated systems *A*, *B*, BG entropy is non-extensive while for a special q-value $S_{q}$ is extensive [32].

Tsallis non-extensive statistical mechanics includes also the q-generalization of the CLT as a q-generalization of the Levy-Gnedenko CLT. This generalization is based at the q-Fourier transform of a q-Gaussian, which can produce an infinite sequence $\left(q_{n}\right)$ of q-parameters [108] in relation with the q-independence of random variables. The q-Fourier transform of q-Gaussian probability functions can be used to the definition of q-independence of random variables. With term q-independence, it means the statistical independency for q=1 but strong correlation for q≠1 [32]. The q-CLT state that an appropriately scaled limit of sums $\left(x_{1},x_{2},\ldots ,x_{N}\right)$ of $q_{k}$ correlated random variables is a $q_{k-1}$ Gaussian random variable corresponding to a statistical attractor. The triplet $\left(q_{k-1},q_{k},q_{k+1}\right)$ Gaussians are connected by the q-Fourier transform and describe three basic processes of non-extensive statistical theory. The $q_{k}$ independent microscopic random variables produce the macroscopic relaxation process. The $q_{k-1}$ Gaussian attractor of the q-CLT describes the entropy production related with the multifractal structure of the dynamical phase space of the system, as the system moves to the optimal q-entropy states. The $q_{k+1}$ Gaussian describes the scale invariance of the q-Gaussian attractor as well as the anomalous diffusion process. In this physical meaning of the generalized q-CLT, Tsallis q-triplet includes three q-parameters $\left(q_{sen},q_{stat},q_{rel}\right)$ corresponding to the triplet $\left(q_{k-1},q_{k},q_{k+1}\right)$ of the q-extended CLT [32,108]. In the case of classical CLT of Boltzmann-Gibbs statistical theory the values of Tsallis q-triplet coincide to the value 1 $\left(q_{sen}=q_{stat}=q_{rel}=1\right)$.

Additionally, and according to Livadiotis and McComas [81], the connection between kappa distributions and Tsallis non-extensive statistical mechanics, is given by the transformation:

(A14) $$\kappa \equiv \frac{1}{q-1}\;\mathrm{or}\;q\equiv 1+\frac{1}{\kappa },$$

where $q\equiv q_{stat}$. The κ-distributions describe the energy distribution function of particles far from thermodynamic equilibrium. The q-Gaussian distributions connected with the dynamics of fractals (fractional-strange dynamics) corresponds asymptotically to the Levy distribution and the random walk on fractal structures [32,56]. The fractional-strange dynamics on fractals producing q-Gaussian distributions is described as stationary solutions of fractional Fokker-Planck-Kolmogorov equations including fractional space and time derivatives $\left(\partial^{\beta }/\partial t^{\beta },\partial^{\alpha }/\partial x^{\alpha }\right)$ [37,54,89].
